# Efficacy and safety of transcatheter aortic valve replacement for the treatment of pure severe native aortic valve regurgitation: a single-arm meta-analysis

**DOI:** 10.3389/fmed.2026.1735206

**Published:** 2026-03-04

**Authors:** Fuli Zhu, Guangyao Zhai, Shunan He, Zijing Liu, Zhe He

**Affiliations:** Department of Cardiology, Beijing Luhe Hospital, Capital Medical University, Beijing, China

**Keywords:** efficacy, meta-analysis, pure severe native aortic valve regurgitation, safety, transcatheter aortic valve replacement

## Abstract

**Objective:**

Numerous studies have reported the efficacy and safety of transcatheter aortic valve replacement (TAVR) for pure severe native aortic valve regurgitation (psNAVR) in recent years; however, these studies show considerable variability in outcomes such as success rate and mortality. Therefore, this meta-analysis was conducted to evaluate the efficacy and safety of TAVR in patients with psNAVR based on the latest research evidence.

**Methods:**

Relevant studies were searched in four databases—PubMed, Embase, Web of Science, and the Cochrane Library—up to August 27, 2025. The primary outcomes were device success, all-cause mortality, and cardiovascular mortality during the perioperative period. Secondary outcomes included perioperative and 1-year post-operative adverse events, such as stroke, acute kidney injury (AKI), new-onset myocardial infarction, major vascular complications, major bleeding events, readmission due to heart failure (HF), and new permanent pacemaker (PPM) implantation. Statistical analyses were performed using Stata 14.0 software.

**Results:**

A total of 29 articles involving 2,773 patients with psNAVR undergoing TAVR were included in the meta-analysis. The device success rate was 87.5% [95% confidence interval (CI): 83.3%−91.2%]. Perioperative all-cause mortality was 3.1% (95% CI: 1.6%−5.1%), and perioperative cardiovascular mortality was 1.4% (95% CI: 0.2%−3.5%). During the perioperative period, the incidence of adverse events was as follows: stroke, 0.7%; AKI, 4.5%; new-onset myocardial infarction, 0.0%; major vascular complications, 3.3%; major bleeding events, 4.4%; and new PPM implantation, 11.4%. At 1 year, the incidence rates were 9.3% for all-cause mortality, 4.3% for cardiovascular mortality, 2.6% for stroke, 9.1% for AKI, 0.0% for new-onset myocardial infarction, 1.6% for major bleeding events, 19.0% for readmission due to HF, and 10.0% for new PPM implantation. Subgroup analysis indicated that geographic location, surgical risk, valve type, and procedural approach influenced the incidence of post-operative adverse events.

**Conclusion:**

TAVR is a valuable therapeutic option for patients with psNAVR at high surgical risk. However, geographic location, surgical risk, valve type, and procedural approach appear to influence the incidence of adverse events after TAVR.

## Highlights

- The device success rate of TAVR for psNAVR treatment was 87.5%.
- TAVR is a valuable therapeutic option for patients with psNAVR who are at high surgical risk.
- Geographic location, surgical risk, valve type, and procedural approach influence the incidence of adverse events after TAVR.
- This study provides comprehensive and objective evidence to support clinical decision-making.

## Introduction

Aortic valve regurgitation (AR) is a common valvular disease (1, 2). Among its subtypes, pure severe native aortic valve regurgitation (psNAVR) is characterized by structural or functional abnormalities of the aortic valve leaflets, leading to retrograde blood flow from the aorta into the left ventricle during diastole (3). The occurrence of psNAVR is closely related to factors such as degenerative changes in the valve leaflets, congenital malformations, and infective endocarditis (4). In the Chinese population, the prevalence of psNAVR is 1.2%, rising to 2% in individuals aged 70 years and older (5). Long-term AR with chronic volume overload increases left ventricular end-diastolic volume and pressure, which initially results in increased left ventricular compliance (6). Although this adaptation allows the left ventricle to accommodate the increased volume, prolonged overload ultimately causes progressive ventricular enlargement, a reduction in left ventricular ejection fraction (LVEF), and elevated left atrial and pulmonary artery wedge pressures, leading to left heart failure (HF) and, in severe cases, biventricular failure (7, 8).

The traditional treatments for AR mainly include drug therapy and surgical aortic valve replacement (9). However, many patients with severe AR are unable to undergo surgery due to factors such as reduced LVEF, advanced age, and multiple comorbidities (10). In recent years, transcatheter aortic valve replacement (TAVR) has emerged as an effective treatment for aortic valve stenosis (11). However, in patients with pure aortic valve regurgitation without valve calcification or stenosis, TAVR still presents considerable technical challenges (12). Although numerous studies in recent years have reported the efficacy and safety of TAVR in psNAVR, the results vary substantially with respect to success rate and mortality. Whether factors such as valve type, procedural approach, and geographic region significantly affect outcomes remains controversial (13–18).

Therefore, this meta-analysis was conducted to summarize the latest evidence and comprehensively evaluate the efficacy and safety of TAVR in patients with psNAVR. This study aims to provide comprehensive and objective evidence to support clinical decision-making.

## Methods

### Search strategy

A systematic literature search was conducted in PubMed, Embase, Web of Science, and the Cochrane Library up to August 27, 2025, with no language restrictions. The search keywords were “aortic valve regurgitation,” “transcatheter aortic valve replacement,” “transcatheter aortic valve implantation,” “TAVR,” and “TAVI.” Keywords within the same category were combined with “OR,” whereas keywords across categories were combined with “AND” (Supplementary Tables S1–S4). In addition, the reference lists of relevant reviews and included articles were screened to identify additional studies eligible for meta-analysis.

### Eligibility criteria

The inclusion criteria were as follows: (1) patients with psNAVR, excluding those with mild or moderate AR or concomitant aortic stenosis; (2) patients with psNAVR undergoing TAVR; (3) studies reporting at least one outcome, such as device success, mortality, or adverse events; and (4) no restriction on study design, with both prospective and retrospective studies eligible. Non-academic publications such as reviews, conference abstracts, commentaries, and letters were excluded. Studies with a sample size of fewer than 10 participants were also excluded to minimize selection bias (19). In cases of duplicate publications or overlapping datasets, only the article with the most complete research information was included.

### Data extraction and quality assessment

Two investigators independently screened the literature according to the inclusion and exclusion criteria. After identifying studies for inclusion in the meta-analysis, the investigators independently extracted data using a predesigned standardized form. The extracted information included the first author, publication year, study region, baseline characteristics of the study population (sample size, age, and sex ratio), aortic valve morphology, surgical risk level, device manufacturer, device type, procedural approach, follow-up duration, and study outcomes. After completing data extraction, the investigators exchanged verification sheets and resolved discrepancies through discussion.

For quality assessment, the Methodological Index for Non-Randomized Studies (MINORS) (20) was applied. The MINORS instrument includes 12 evaluation items; however, for single-arm studies without a control group, only the first eigt items were assessed. Each item was scored from 0 to 2, with a maximum total of 16 points. A score of 0 indicated “not reported,” 1 indicated “reported but inadequate,” and 2 indicated “reported and adequate.” Studies were categorized as having low (0–5), moderate (6–11), or high (12–16) methodological quality.

### Statistical analysis

Statistical analyses were performed using Stata 14.0 software (StataCorp). The primary outcomes were device success, perioperative all-cause mortality, and perioperative cardiovascular mortality, where the perioperative period was defined as during surgery and within 30 days post-operatively. Secondary outcomes included perioperative and 1-year post-operative adverse events, such as stroke, acute kidney injury (AKI), new-onset myocardial infarction, major vascular complications, major bleeding events, readmission due to HF, and new permanent pacemaker (PPM) implantation.

All study outcomes were categorical variables, and incidence rate (IR) with 95% confidence interval (CI) was used as the effect size (ES). Heterogeneity was assessed using Cochran's *Q* test and the *I*^2^ statistic (21). A *Q* test *P*-value < 0.05 or *I*^2^ > 50% indicated significant heterogeneity; otherwise, heterogeneity was considered non-significant. Because of notable heterogeneity in study design, treatment protocols, and device types among the included studies, a random-effects model was applied to synthesize the ES.

For perioperative outcomes, subgroup analyses were conducted to examine the effects of study design, geographic region, surgical risk, valve type, procedural approach, generation of devices, and risk of bias on heterogeneity and pooled ES. Through sensitivity analysis, studies with a significant impact on heterogeneity were excluded, and the difference between the pooled ES of the remaining studies and the original meta-analysis result was compared. Publication bias was evaluated using the Egger test and funnel plots (22).

## Results

### Study selection

**Figure 1:**
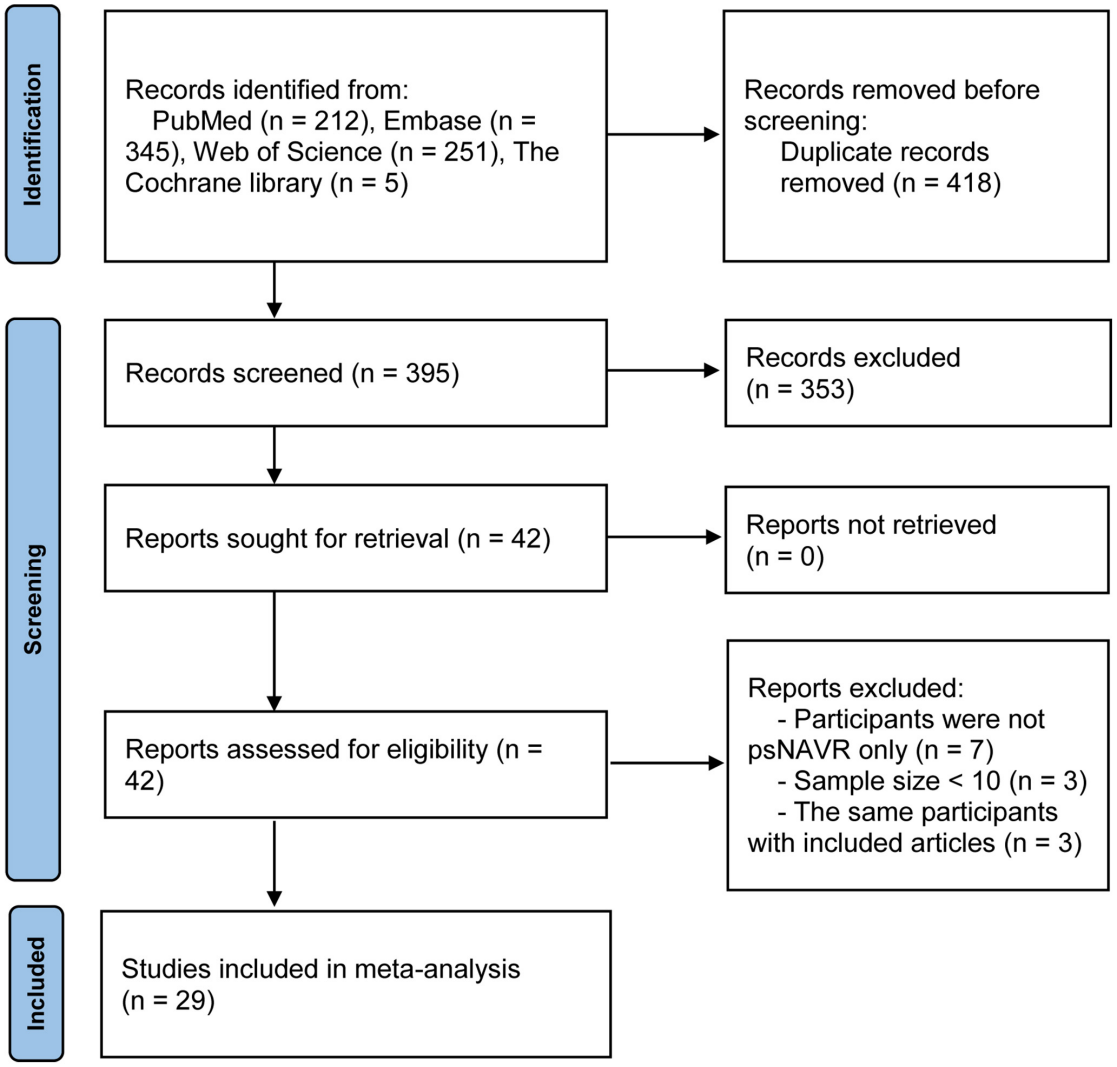
Flow diagram of the study selection process.

A total of 813 articles were identified through the initial search. Of these, 418 were removed as duplicates. After screening titles and abstracts, 353 articles were excluded, and 42 articles were retrieved for full-text eligibility assessment. Ultimately, 29 articles (4, 13–18, 23–44) met the inclusion criteria for the meta-analysis (Figure 1).

### Study characteristics and study quality

The 29 included studies involved 2,773 patients with psNAVR undergoing TAVR, with sample sizes ranging from 10 to 598 participants. Among them, six were prospective studies (13, 30, 35, 37, 38, 42), and the remainder were retrospective. Apart from a few multinational collaborative studies (15, 17, 31–33, 41), most were conducted in China, Germany, Italy, France, and the United States. The characteristics of these studies are summarized in Table 1.

**Table 1:** Characteristics of the 29 included studies.

**Study**	**Location**	**Design**	**Follow-up months**	**AV morphology**	**Surgical risk**	**Device**	**Type of valve**	**Approach**	***n*, M/F**	**Age, years**	**NYHA III/IV, *n* (%)**
Chen, J 2025	China	RS	18	Bicuspid valve	High risk only	J-Valve	Third-generation AR-specific valve	TA	23, 20/3	71 ± 11	15 (65.22)
					High risk only	VitaFlow	Second -generation valve	TF	25, 19/6	70 ± 7	8 (32.00)
Garcia, S 2023	USA	RS	12	Tricuspid valve (89%)	Not High risk only	J-Valve	Third-generation AR-specific valve	TF (21/27)	27, 16/11	81 (72–85)	26 (96.0)
Hinkov, H 2024	Germany	RS	12	Tricuspid valve	Not High risk only	JenaValve Trilogy	Third-generation AR-specific valve	TF (25/27)	27, 18/9	65.3 (36.8, 77.6)	NR
Kong, XQ 2024	China	RS	12	Tricuspid valve	High risk only	VitaFlow	Second -generation valve	TF	62, 36/26	71.56 ± 7.34	38 (61.3)
Le Ruz, R 2024	France	PS	12	Bicuspid or Tricuspid valve	Not High risk only	CoreValve Evolut, SAPIEN 3	Second -generation valve	TF (207/227)	227, 146/81	81.0 (73.5–85.0)	150 (77.3)
Lin, DW 2024	China	RS	6	Bicuspid or Tricuspid valve	Not High risk only	Venus-A, VitaFlow	First-generation valve	TF	103, 62/41	72.1 ± 8.1	87 (84.5)
Liu, H 2018	China	RS	12	Tricuspid valve (95.3%)	High risk only	J-Valve	Third-generation AR-specific valve	TA	43, 30/13	73.9 ± 5.7	14 (32.6)
Liu, L 2022	China	RS	12	Tricuspid valve (93.3%)	High risk only	J-Valve	Third-generation AR-specific valve	TA	134, 100/34	73.1 ± 6.4	131 (97.8)
Mao, Y 2024	China	RS	24	Bicuspid or Tricuspid valve	Not High risk only	J-Valve	Third-generation AR-specific valve	TA	598, 449/149	72.0 (66.0, 78.0)	579 (96.8)
Orzalkiewicz, M 2024	Italy	RS	12	Tricuspid valve	Not High risk only	Sapien 3	Second -generation valve	TF	13, 9/4	80.8 ± 5.6	NR
Pan, W 2025	China	PS	1	Bicuspid or Tricuspid valve	Not High risk only	Hanchor Valve	Third-generation AR-specific valve	TF	128, 98/30	74 (70, 78)	108 (84.4)
Poletti, E 2023	Europe and USA	RS	12	Bicuspid or Tricuspid valve	High risk only	THVs	SEV or BEV	TF (192/201)	201, 111/90	79 (73, 83)	144 (76.2)
Purita, P 2020	Europe	RS	12	Tricuspid valve (96%)	Not High risk only	ACURATE neo THV	Second -generation valve	TF	24, 10/14	79.4 (50–88)	23 (95.8)
Roy, DA 2013	Worldwide	RS	12	Bicuspid or Tricuspid valve	High risk only	CoreValve	First-generation valve	TF (35/43)	43, 20/23	75.3 ± 8.8	42 (97.7)
Sawaya, FJ 2017	Worldwide	RS	1	Bicuspid or Tricuspid valve	Not High risk only	CoreValve, Evolut R, SAPIEN XT, SAPIEN 3, Lotus Valve System, Direct Flow, JenaValve	First/second-generation valve, third-generation AR-specific valve	TF (51/78)	78, 46/32	74 ± 10	NR
Schlingloff, F 2014	Germany	RS	12	Bicuspid or Tricuspid valve	High risk only	JenaValve	Third-generation AR-specific valve	TA	10, 6/4	82 (59–90)	9 (90.0)
Schofer, J 2015	Europe	RS	1	Bicuspid or Tricuspid valve	High risk only	Direct Flow	Second -generation valve	TF	11, 4/7	78 (46–85)	9 (88.9)
Seiffert, M 2014	Germany	RS	6	Bicuspid or Tricuspid valve	High risk only	JenaValve	Third-generation AR-specific valve	TA	31, 20/11	73.8 ± 9.1	12 (38.7)
Shi, J 2020	China	PS	24	Tricuspid valve (95.5%)	High risk only	J-Valve	Third-generation AR-specific valve	TA	44, 30/14	76.2 ± 5.5	44 (100)
Testa, L 2014	Italy	RS	12	Bicuspid or Tricuspid valve	High risk only	CoreValve	First-generation valve	TF (21/26)	26, 16/10	73 ± 10	25 (95)
Vahl, TP 2024	USA	PS	12	Tricuspid valve	High risk only	JenaValve	Third-generation AR-specific valve	TF	180, 95/85	75.5 ± 10.8	122 (68)
Wang, Y 2025	China	PS	60	Tricuspid valve (97.2%)	High risk only	J-Valve	Third-generation AR-specific valve	TA	36, 25/11	73.78 ± 5.96	35 (97.22)
Yang, L 2025	China	RS	1	Tricuspid valve (94%)	Not High risk only	Venus A or VitaFlow valve	First-generation valve	TF	87, 41/46	45 74.4 ± 8.4; 42 69.7 ± 7.8	38 (43.68)
Yin, WH 2022	China	RS	14	Bicuspid or Tricuspid valve	High risk only	CoreValve, Lotus, and Spien XT	SEV	TF	15, 11/4	72.0 ± 17.2	12 (80)
					High risk only	Evolut R, J-valve	SEV	TF	10, 7/3	72.8 ± 11.7	6 (60)
Yoon, SH 2017	Worldwide	RS	12	Bicuspid or Tricuspid valve	Not High risk only	CoreValve, Lotus, Spien XT, Evolut R, J-valve, et. al	SEV or BEV	TF (233/331)	331, 172/159	74.4 ± 12.2	293 (88.5)
Yu, FC 2025	China	PS	1	Bicuspid or Tricuspid valve	Not High risk only	VitaFlow	Second -generation valve	TF	100, NR	72.7 ± 7.2	NR
Zheng, HJ 2023	China	RS	12	Tricuspid valve (97.8%)	High risk only	Venus-A	First-generation valve	TF	45, 33/12	73.5 ± 5.5	43 (95.6)
Zhu, L 2018	China	RS	6	Tricuspid valve	High risk only	J-Valve	Third-generation AR-specific valve	TA	44, 31/13	73.8 ± 5.6	43 (100)
Zhu, P 2025	China	RS	6	Tricuspid valve (87.1%)	Not High risk only	J-Valve	Third-generation AR-specific valve	TA	47, 35/12	73.0 ± 9.0	46 (97.9)

AV, aortic valve; BEV, Balloon-Expandable valve; F, female; M, male; NR, not reached; NYHA, New York Heart Association; PS, Prospective study; RS, Retrospective study; SEV, self-expandable valve; TA, transapical; TF, transfemoral; USA, the United States of America.

The MINORS scores of the included studies ranged from 9 to 15. Thirteen studies scored 12 or higher and were classified as low risk of bias (13, 14, 25, 29, 35–38, 40–43), while the remaining studies were classified as moderate risk of bias (Supplementary Table S5).

### Meta-analysis

#### Primary outcomes

**Figure 2:**
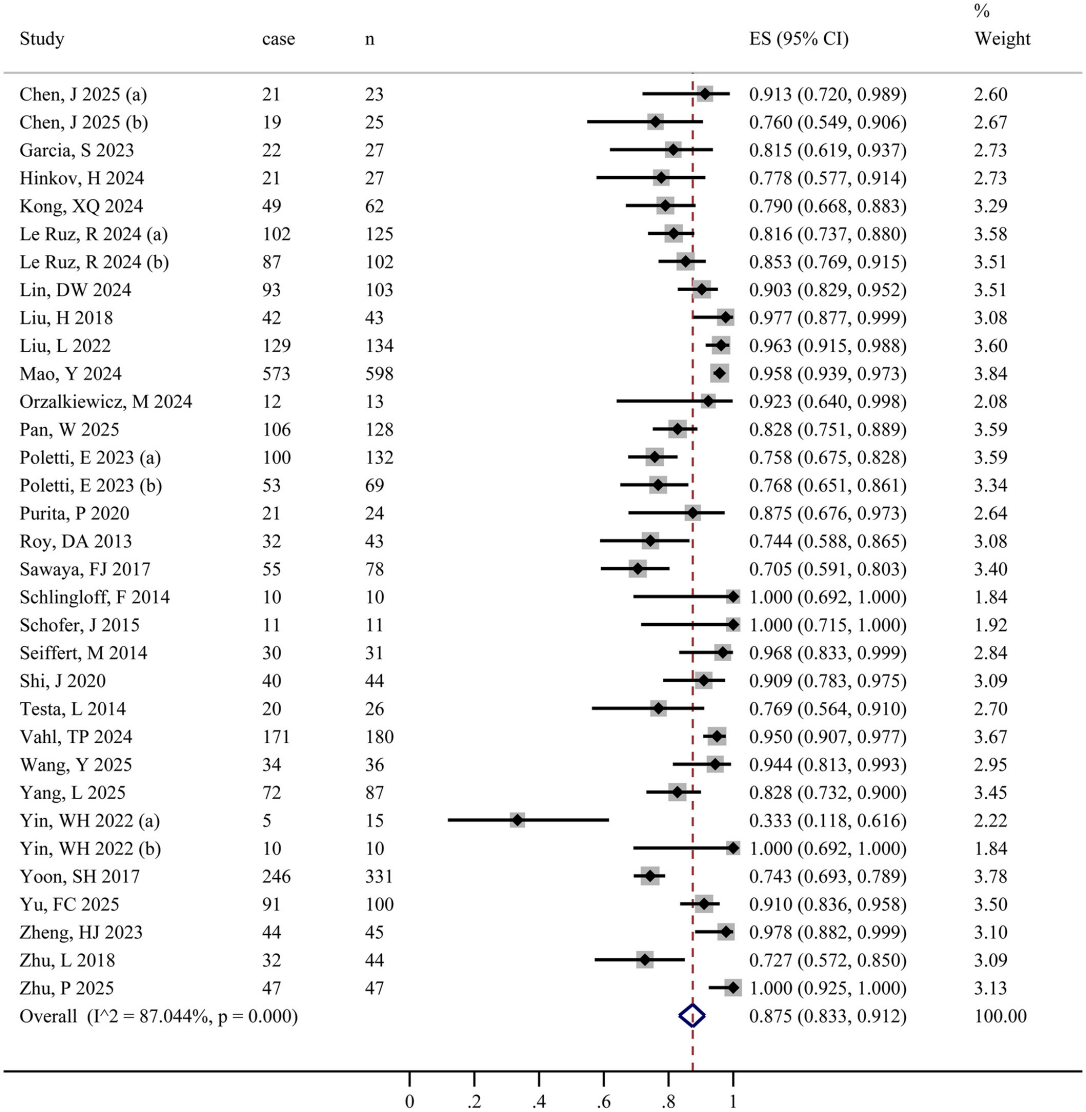
Forest plot of device success. The size of the black squares is proportional to the weight of each study in the pooled estimate calculated using a random-effects model. The diamond below the studies represents the pooled effect estimate, with its center and vertical line (red) indicating the overall event rate and its lateral edges denoting the 95% CI.

Because four studies stratified participants by specific clinical features and reported results separately, a total of 33 datasets were available. Figure 2 presents the forest plot of device success, including 33 datasets and 2,773 participants (Table 2). The pooled random-effects model yielded an IR of 0.875 (95% CI: 0.833–0.912), indicating a device success rate of 87.5% (95% CI: 83.3%−91.2%). Significant heterogeneity was observed among the included studies (*I*^2^ = 87.0%, *P* < 0.001).

**Table 2:** Summary of perioperative outcomes.

**Outcomes**	**No. of study**	**Sample size**	**Incidence rate (95%CI)**	Heterogeneity test		**Egger' s test *P* value**
				***P*** **value**	*I*^2^ **(%)**	
Device success	33	2,773	0.875 (0.833, 0.912)	<0.001	87.0	0.367
Total mortality	31	2,139	0.031 (0.016, 0.051)	<0.001	71.2	0.349
Cardiovascular disease mortality	19	1,589	0.014 (0.002, 0.035)	<0.001	69.3	0.058
Stroke	19	1,953	0.007 (0.001, 0.017)	0.034	40.7	0.891
Acute kidney injury	18	1,977	0.045 (0.022, 0.074)	<0.001	79.6	0.683
New-onset Myocardial infarction	16	1,361	0.000 (0.000, 0.001)	0.934	0.0	0.892
Major vascular complication	19	1,916	0.033 (0.010, 0.066)	<0.001	84.9	0.461
Major bleeding events	21	2,025	0.044 (0.018, 0.076)	0.001	83.9	0.333
New permanent pacemaker implantation	27	2,411	0.114 (0.084, 0.147)	<0.001	76.2	0.741

**Figure 3:**
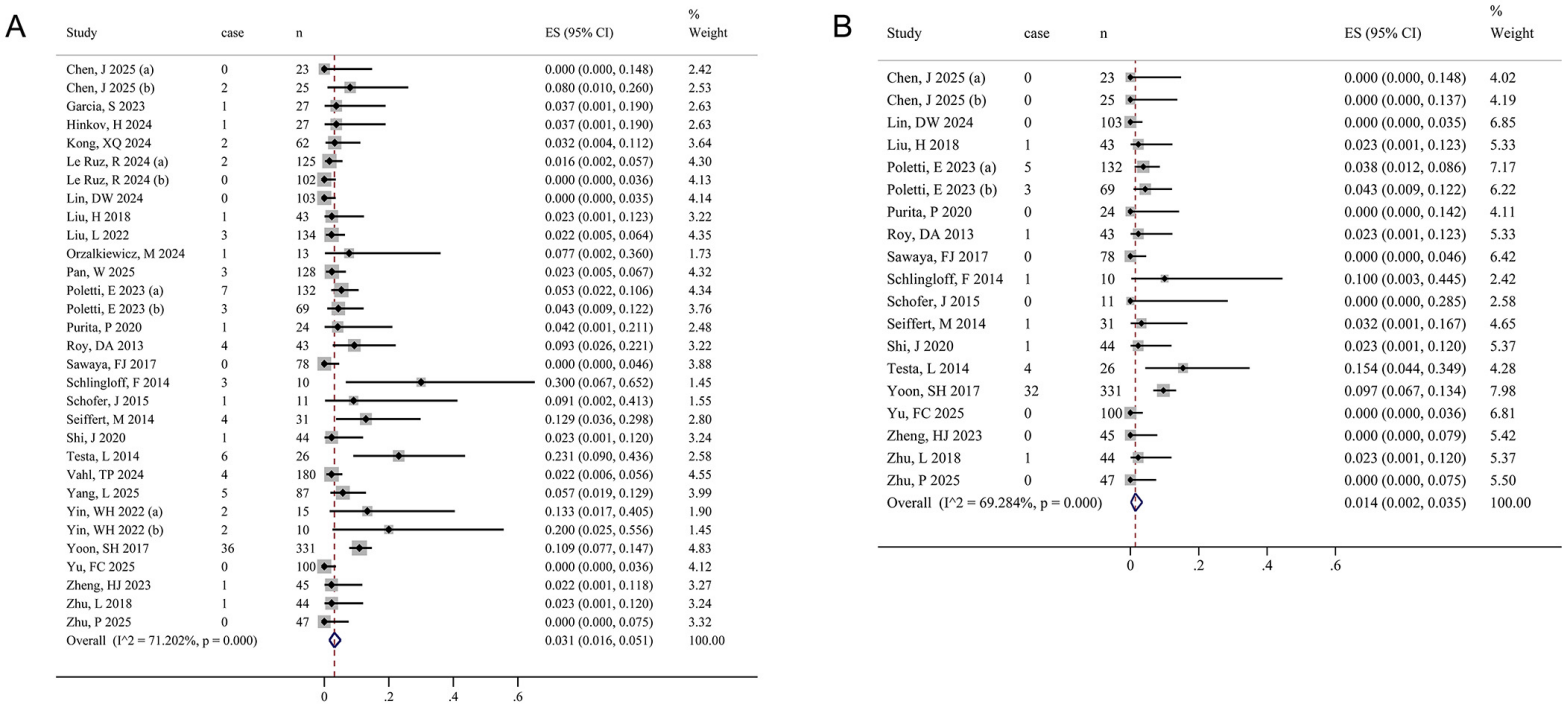
Forest plots of perioperative mortality outcomes. **(A)** All-cause mortality. **(B)** Cardiovascular mortality. The size of the black squares is proportional to the weight of each study in the pooled estimate calculated using a random-effects model. The diamond below the studies represents the pooled effect estimate, with its center and vertical line (red) indicating the overall event rate and its lateral edges denoting the 95% CI.

A total of 2,139 participants contributed data on perioperative all-cause mortality (Figure 3A, Table 2). The pooled estimate was IR = 0.031 (95% CI: 0.016–0.051), with significant heterogeneity (*I*^2^ = 71.2%, *P* < 0.001). In addition, 1,589 participants provided data on perioperative cardiovascular mortality (Figure 3B, Table 2). The pooled estimate was IR = 0.014 (95% CI: 0.002–0.035), with significant heterogeneity (*I*^2^ = 69.3%, *P* < 0.001).

#### Secondary perioperative outcomes

**Figure 4:**
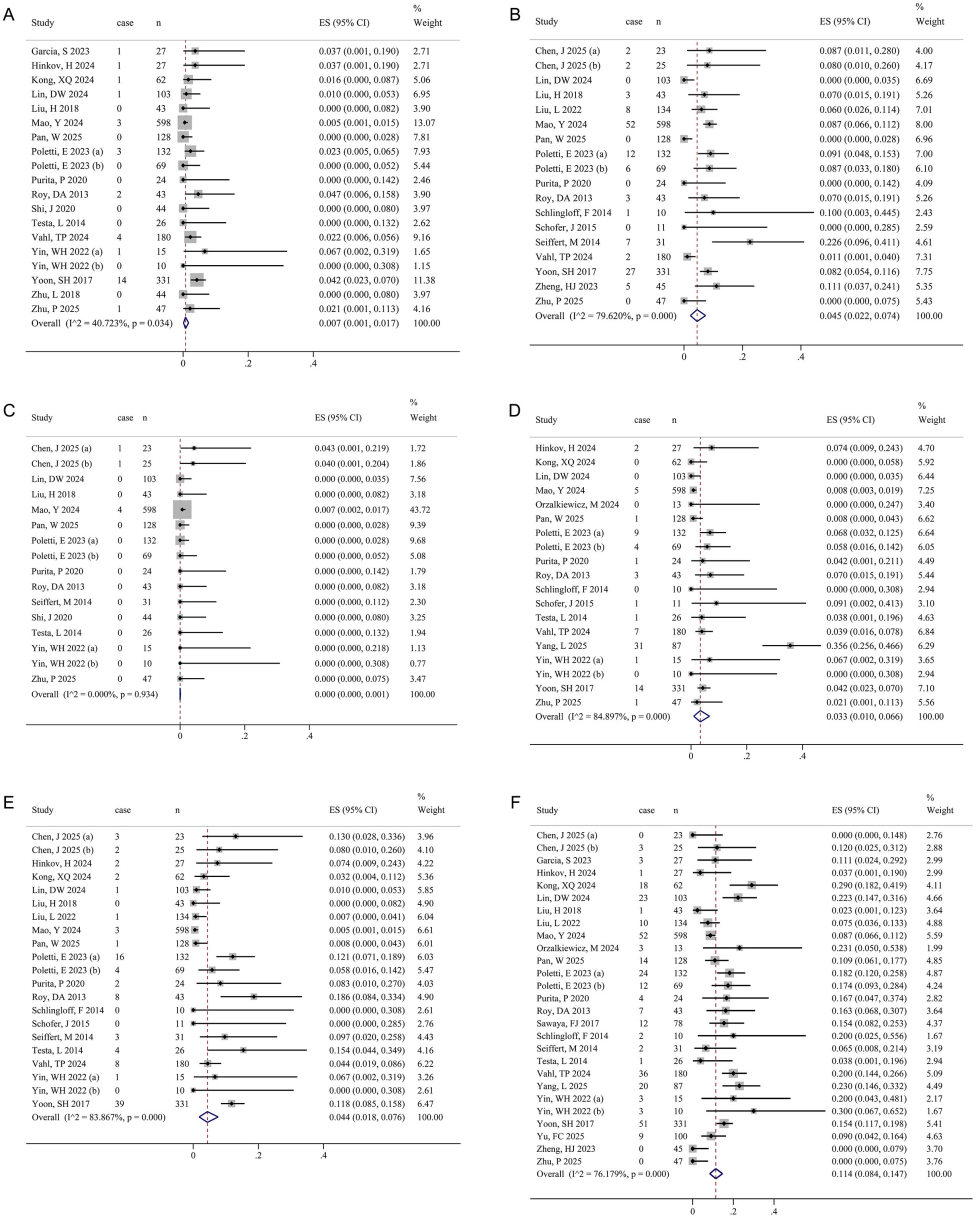
Forest plots of perioperative adverse events. **(A)** Stroke. **(B)** Acute kidney injury. **(C)** New-onset myocardial infarction. **(D)** Major vascular complications. **(E)** Major bleeding events. **(F)** New permanent pacemaker implantation. The size of the black squares is proportional to the weight of each study in the pooled estimate calculated using a random-effects model. The diamond below the studies represents the pooled effect estimate, with its center and vertical line (red) indicating the overall event rate and its lateral edges denoting the 95% CI.

The pooled results for stroke, AKI, new-onset myocardial infarction, major vascular complications, major bleeding events, and new PPM implantation are shown in Figures 4A–F and Table 2. The random-effects model indicated that, for TAVR in psNAVR, the perioperative incidences were 0.7% for stroke, 4.5% for AKI, 0.0% for new-onset myocardial infarction, 3.3% for major vascular complications, 4.4% for major bleeding events, and 11.4% for new PPM implantation. Except for new-onset myocardial infarction, all outcomes demonstrated significant heterogeneity (*I*^2^ > 50%, *P* < 0.05).

#### Secondary 1-year post-operative outcomes

The pooled results for all-cause mortality, cardiovascular mortality, stroke, AKI, new-onset myocardial infarction, major bleeding events, readmission due to HF, and new PPM implantation at 1 year are shown in Supplementary Figures S1–S8, and Table 3. The random-effects model indicated incidence rates of 9.3% for all-cause mortality, 4.3% for cardiovascular mortality, 2.6% for stroke, 9.1% for AKI, 0.0% for new-onset myocardial infarction, 1.6% for major bleeding events, 19.0% for readmission due to HF, and 10.0% for new PPM implantation. Significant heterogeneity was observed for all-cause mortality, cardiovascular mortality, readmission due to HF, and new PPM implantation (*I*^2^ > 50% or *P* < 0.05). For the remaining outcomes, no significant heterogeneity was found (*I*^2^ = 0%, *P* > 0.05).

**Table 3:** Summary of 1-year post-operative outcomes.

**Outcomes**	**No. of study**	**Sample size**	**Incidence rate (95%CI)**	Heterogeneity test		**Egger' s test *P* value**
				***P*** **value**	*I*^2^ **(%)**	
Total mortality	16	895	0.093 (0.065, 0.126)	0.026	45.1	0.058
Cardiovascular disease mortality	9	342	0.043 (0.012, 0.088)	0.021	55.8	0.714
Stroke	4	199	0.026 (0.005, 0.056)	0.911	0.0	0.051
Acute kidney injury	2	120	0.091 (0.044, 0.151)	NA	NA	NA
New-onset myocardial infarction	6	328	0.000 (0.000, 0.010)	0.459	0.0	0.350
Major bleeding events	4	164	0.016 (0.000, 0.047)	0.413	0.0	0.536
Readmission due to heart failure	6	148	0.190 (0.066, 0.351)	0.001	75.2	0.156
New permanent pacemaker implantation	6	327	0.100 (0.029, 0.200)	<0.001	81.7	0.861

### Subgroup analysis

Table 4 summarizes the subgroup analyses of perioperative outcomes. Because of the extremely low incidence of new-onset myocardial infarction, no subgroup analysis was performed for this outcome. For device success, cardiovascular mortality, and stroke, and major bleeding events, geographic location, valve type, and procedural approach significantly influenced the pooled ES (*P* < 0.05). For major bleeding events, geographic location, valve type, generation of devices, and procedural approach significantly influenced the pooled ES (*P* < 0.05). For all-cause mortality, study design, surgical risk, and valve type were significant influencing factors (*P* < 0.05). For AKI, study design, surgical risk, valve type, and procedural approach were significant influencing factors (*P* < 0.05). For major vascular complications and new PPM implantation, procedural approach was the influencing factor (*P* < 0.05). For new PPM implantation, procedural approach, and generation of devices were the influencing factors (*P* < 0.05). Geographic location and procedural approach also contributed to heterogeneity in stroke incidence after surgery. No significant influencing factors were found for the remaining outcomes.

**Table 4:** Outcomes of subgroup analyses of perioperative outcomes.

**Outcomes**	**No. of studies**	**Incidence rate (95%CI)**	Heterogeneity test	
			***P*** **value**	*I*^2^ **(%)**
**Device success**				
Total	33	0.875 (0.833, 0.912)	<0.001	87.0
**Location**		<0.001		
China	17	0.900 (0.849, 0.942)	<0.001	85.0
Western	13	0.870 (0.811, 0.920)	<0.001	73.2
Worldwide	3	0.738 (0.696, 0.778)	NA	NA
**Design**		0.443		
RS	26	0.870 (0.814, 0.917)	<0.001	88.9
PS	7	0.889 (0.839, 0.931)	0.002	71.5
**Surgical risk**			0.773	
High risk only	19	0.883 (0.821, 0.934)	<0.001	83.3
Not high risk only	14	0.865 (0.801, 0.919)	<0.001	90.6
**Type of valve**			<0.001	
SEV	27	0.892 (0.849, 0.929)	<0.001	84.0
BEV	4	0.833 (0.788, 0.874)	0.458	0.0
SEV or BEV	2	0.737 (0.693, 0.779)	NA	NA
**Approach**			<0.001	
TA	10	0.952 (0.913, 0.981)	0.001	68.0
TF	13	0.881 (0.819, 0.933)	<0.001	78.2
Multi-approach	10	0.773 (0.744, 0.801)	0.395	4.9
**Risk of bias**			0.896	
Moderate	19	0.872 (0.818, 0.919)	<0.001	80.8
Low	14	0.877 (0.808, 0.934)	<0.001	91.0
**Generation of devices**			0.127	
First	3	0.853 (0.642, 0.984)	NA	NA
Second	8	0.861 (0.813, 0.903)	0.153	34.5
CADE	16	0.908 (0.858, 0.950)	<0.001	86.0
Multiple	6	0.785 (0.671, 0.882)	<0.001	85.7
**Total mortality**				
Total	31	0.031 (0.016, 0.051)	<0.001	71.2
**Location**			0.164	
China	15	0.017 (0.004, 0.034)	0.024	44.7
Western	13	0.044 (0.016, 0.082)	<0.001	67.6
Worldwide	3	0.051 (0.000, 0.167)	NA	NA
**Design**			0.001	
RS	25	0.042 (0.022, 0.068)	<0.001	67.7
PS	6	0.011 (0.002, 0.023)	0.227	27.7
**Surgical risk**			0.045	
High risk only	18	0.046 (0.024, 0.072)	0.007	51.0
Not high risk only	13	0.016 (0.001, 0.044)	<0.001	81.8
**Type of valve**			0.033	
SEV	25	0.032 (0.016, 0.053)	<0.001	60.5
BEV	4	0.015 (0.000, 0.054)	0.056	60.3
SEV or BEV	2	0.075 (0.051, 0.103)	NA	NA
**Approach**			0.851	
TA	8	0.026 (0.003, 0.064)	0.030	54.9
TF	13	0.023 (0.005, 0.049)	0.005	57.2
Multi-approach	10	0.042 (0.012, 0.085)	<0.001	82.8
**Risk of bias**			0.655	
Moderate	19	0.028 (0.012, 0.049)	0.003	54.3
Low	12	0.037 (0.008, 0.078)	<0.001	82.7
**Generation of devices**			0.370	
First	3	0.095 (0.011, 0.231)	NA	NA
Second	8	0.013 (0.000, 0.042)	0.028	55.4
CADE	14	0.026 (0.012, 0.043)	0.135	30.2
Multiple	6	0.042 (0.000, 0.122)	<0.001	88.3
**Cardiovascular disease mortality**				
Total	19	0.014 (0.002, 0.035)	<0.001	69.3
**Location**			0.004	
China	9	0.001 (0.000, 0.010)	0.663	0.0
Western	7	0.032 (0.009, 0.065)	0.322	14.1
Worldwide	3	0.030 (0.000, 0.124)	NA	NA
**Design**			0.108	
RS	17	0.016 (0.002, 0.039)	<0.001	68.1
PS	2	0.002 (0.002, 0.022)	NA	NA
**Surgical risk**			0.236	
High risk only	13	0.022 (0.009, 0.039)	0.472	0.0
Not high risk only	6	0.006 (0.000, 0.049)	<0.001	89.3
**Type of valve**			0.002	
SEV	16	0.008 (0.000, 0.023)	0.073	36.4
BEV	1	0.043 (0.009, 0.122)	NA	NA
SEV or BEV	2	0.067 (0.044, 0.094)	NA	NA
**Approach**			0.004	
TA	7	0.012 (0.000, 0.036)	0.639	0.0
TF	6	0.000 (0.000, 0.002)	0.980	0.0
Multi-approach	6	0.044 (0.012, 0.092)	<0.001	78.3
**Risk of bias**			0.428	
Moderate	14	0.008 (0.000, 0.020)	0.304	13.7
Low	5	0.033 (0.000, 0.107)	<0.001	87.4
**Generation of devices**			0.085	
First	3	0.035 (0.000, 0.144)	NA	NA
Second	4	0.000 (0.000, 0.006)	0.895	0.0
CADE	9	0.021 (0.007, 0.040)	0.725	0.0
Multiple	3	0.017 (0.000, 0.112)	NA	NA
**Stroke**				
Total	19	0.007 (0.001, 0.017)	0.034	40.7
**Location**			<0.001	
China	10	0.000 (0.000, 0.003)	0.592	0.0
Western	7	0.011 (0.002, 0.025)	0.632	0.0
Worldwide	2	0.039 (0.021, 0.063)	NA	NA
**Design**			0.327	
RS	16	0.009 (0.001, 0.021)	0.044	41.1
PS	3	0.005 (0.000, 0.026)	NA	NA
**Surgical risk**			0.956	
High risk only	11	0.007 (0.001, 0.018)	0.608	0.0
Not High risk only	8	0.010 (0.000, 0.029)	0.003	67.7
**Type of valve**			0.001	
SEV	16	0.004 (0.000, 0.011)	0.385	6.0
BEV	2	0.000 (0.000, 0.009)	NA	NA
SEV or BEV	1	0.042 (0.023, 0.070)	NA	NA
**Approach**			0.005	
TA	5	0.001 (0.000, 0.006)	0.781	0.0
TF	7	0.003 (0.000, 0.014)	0.369	7.8
Multi-approach	7	0.022 (0.008, 0.040)	0.297	17.5
**Risk of bias**			0.571	
Moderate	11	0.006 (0.000, 0.017)	0.327	12.3
Low	8	0.008 (0.000, 0.027)	0.009	62.8
**Generation of devices**			0.121	
First	2	0.021 (0.000, 0.077)	NA	NA
Second	2	0.008 (0.000, 0.046)	NA	NA
CADE	11	0.005 (0.000, 0.013)	0.222	23.3
Multiple	4	0.017 (0.002, 0.041)	0.313	15.8
**Acute kidney injury**				
Total	18	0.045 (0.022, 0.074)	<0.001	79.6
**Location**			0.331	
China	9	0.037 (0.008, 0.081)	<0.001	84.9
Western	7	0.051 (0.007, 0.120)	<0.001	77.6
Worldwide	2	0.077 (0.051, 0.107)	NA	NA
**Design**			<0.001	
RS	16	0.056 (0.033, 0.084)	<0.001	66.3
PS	2	0.005 (0.000, 0.017)	NA	NA
**Surgical risk**			0.032	
High risk only	12	0.068 (0.036, 0.107)	0.003	60.8
Not high risk only	6	0.017 (0.000, 0.060)	<0.001	90.8
**Type of valve**			0.003	
SEV	15	0.046 (0.020, 0.080)	<0.001	76.0
BEV	2	0.014 (0.001, 0.037)	NA	NA
SEV or BEV	1	0.082 (0.054, 0.116)	NA	NA
**Approach**			<0.001	
TA	7	0.067 (0.030, 0.114)	0.013	63.0
TF	7	0.009 (0.000, 0.040)	0.005	68.0
Multi-approach	4	0.081 (0.060, 0.106)	0.976	0.0
**Risk of bias**			0.651	
Moderate	14	0.040 (0.011, 0.080)	<0.001	76.6
Low	4	0.062 (0.026, 0.112)	<0.001	85.7
**Generation of devices**			0.444	
First	2	0.090 (0.036, 0.162)	NA	NA
Second	3	0.016 (0.000, 0.087)	NA	NA
CADE	11	0.050 (0.020, 0.091)	<0.001	83.1
Multiple	2	0.051 (0.032, 0.074)	NA	NA
**Major vascular complication**				
Total	19	0.033 (0.010, 0.066)	<0.001	84.9
**Location**			0.759	
China	8	0.026 (0.000, 0.098)	<0.001	93.0
Western	9	0.038 (0.020, 0.060)	0.906	0.0
Worldwide	2	0.042 (0.022, 0.066)	NA	NA
**Design**			0.169	
RS	17	0.037 (0.009, 0.077)	<0.001	86.2
PS	2	0.024 (0.009, 0.045)	NA	NA
**Surgical risk**			0.926	
High risk only	10	0.030 (0.013, 0.053)	0.313	14.1
Not high risk only	9	0.036 (0.003, 0.095)	<0.001	92.2
**Type of valve**			0.660	
SEV	15	0.038 (0.007, 0.086)	<0.001	87.7
BEV	3	0.015 (0.000, 0.062)	NA	NA
SEV or BEV	1	0.042 (0.023, 0.070)	NA	NA
**Approach**			<0.001	
TA	3	0.000 (0.000, 0.005)	NA	NA
TF	10	0.032 (0.000, 0.108)	<0.001	89.9
Multi-approach	6	0.047 (0.030, 0.066)	0.778	0.0
**Risk of bias**			0.153	
Moderate	11	0.052 (0.008, 0.121)	<0.001	88.0
Low	8	0.012 (0.000, 0.033)	0.011	61.5
**Generation of devices**			0.555	
First	2	0.057 (0.010, 0.129)	NA	NA
Second	4	0.008 (0.000, 0.063)	0.195	36.1
CADE	8	0.024 (0.006, 0.052)	0.001	70.4
Multiple	5	0.060 (0.000, 0.217)	<0.001	94.5
**Major bleeding events**				
Total	21	0.044 (0.018, 0.076)	<0.001	83.9
**Location**			<0.001	
China	10	0.009 (0.000, 0.026)	0.024	52.9
Western	9	0.065 (0.037, 0.098)	0.235	23.4
Worldwide	2	0.122 (0.090, 0.158)	NA	NA
**Design**			0.077	
RS	19	0.048 (0.018, 0.088)	<0.001	84.9
PS	2	0.026 (0.010, 0.048)	NA	NA
**Surgical risk**			0.425	
High risk only	15	0.050 (0.022, 0.087)	<0.001	64.6
Not high risk only	6	0.034 (0.001, 0.098)	<0.001	93.1
**Type of valve**			<0.001	
SEV	18	0.042 (0.015, 0.077)	<0.001	79.6
BEV	2	0.020 (0.003, 0.046)	NA	NA
SEV or BEV	1	0.118 (0.085, 0.158)	NA	NA
**Approach**			<0.001	
TA	6	0.012 (0.000, 0.049)	0.005	69.8
TF	9	0.017 (0.003, 0.038)	0.243	22.5
Multi-approach	6	0.110 (0.085, 0.139)	0.392	3.9
**Risk of bias**			0.988	
Moderate	14	0.043 (0.014, 0.083)	<0.001	73.8
Low	7	0.044 (0.003, 0.114)	<0.001	91.8
**Generation of devices**			0.010	
First	2	0.173 (0.090, 0.274)	NA	NA
Second	4	0.039 (0.007, 0.087)	0.573	0.0
CADE	11	0.031 (0.007, 0.067)	<0.001	82.8
Multiple	4	0.038 (0.000, 0.136)	<0.001	83.4
**New permanent pacemaker implantation**				
Total	27	0.114 (0.084, 0.147)	<0.001	76.2
**Location**			0.187	
China	14	0.094 (0.053, 0.144)	<0.001	83.3
Western	10	0.140 (0.100, 0.185)	0.158	31.3
Worldwide	3	0.153 (0.120, 0.188)	NA	NA
**Design**			0.738	
RS	24	0.111 (0.078, 0.149)	<0.001	77.0
PS	3	0.133 (0.073, 0.206)	NA	NA
**Surgical risk**			0.908	
High risk only	15	0.110 (0.063, 0.165)	<0.001	77.0
Not high risk only	12	0.118 (0.080, 0.162)	<0.001	76.6
**Type of valve**			0.312	
SEV	22	0.104 (0.069, 0.145)	<0.001	79.4
BEV	3	0.136 (0.079, 0.204)	NA	NA
SEV or BEV	2	0.153 (0.119, 0.190)	NA	NA
**Approach**			<0.001	
TA	7	0.042 (0.013, 0.083)	0.016	61.7
TF	12	0.156 (0.101, 0.218)	<0.001	75.1
Multi-approach	8	0.144 (0.116, 0.175)	0.354	9.8
**Risk of bias**			0.576	
Moderate	17	0.107 (0.070, 0.150)	<0.001	71.5
Low	10	0.125 (0.074, 0.185)	<0.001	82.9
**Generation of devices**			0.004	
First	3	0.047 (0.000, 0.190)	NA	NA
Second	5	0.167 (0.082, 0.272)	0.023	64.7
CADE	13	0.085 (0.051, 0.126)	<0.001	76.2
Multiple	6	0.176 (0.140, 0.214)	0.325	14.0

BEV, Balloon-Expandable valve; CAED, contemporary anchoring-enabled devices; NA, not available; PS, Prospective study; RS, Retrospective study; SEV, self-expandable valve; TA, transapical; TF, transfemoral.

### Sensitivity analysis

The sensitivity analysis results of device success, all-cause mortality, cardiovascular mortality, stroke, AKI, major vascular complications, major bleeding events, and new PPM implantation of perioperative were shown in Supplementary Figures S9–S15. After excluding 13 studies (14, 15, 18, 27, 28, 31, 33, 37, 40, 41, 43, 44), the *I*^2^ of device success dropped to 36.5%, and the pooled ES was 87.6% (95% CI: 84.5%−90.4%). Following the exclusion of seven studies (15, 16, 26, 34, 36, 41, 42), the *I*^2^ of all-cause mortality fell to 27.4%, corresponding to a pooled ES of 2.4% (95% CI: 1.3%−3.7%). Exclusion of two studies (36, 41) reduced the *I*^2^ for cardiovascular mortality to 16.7%, and the pooled ES was 0.6% (95% CI: 0.0%−1.6%). After excluding one study (41), the *I*^2^ of stroke dropped to 12.1%, and the pooled ES was 0.4% (95% CI: 0.0%−1.0%). After excluding three studies (26, 30, 37), the *I*^2^ of AKI dropped to 16.7%, and the pooled ES was 6.7% (95% CI: 4.8%−8.9%). Exclusion of four studies (14, 31, 39, 41), the *I*^2^ of major vascular complications fell to 36.3%, corresponding to a pooled ES of 1.6% (95% CI: 0.3%−3.5%). After excluding six studies (14, 23, 31, 33, 36, 41), the *I*^2^ of major bleeding events dropped to 35.4%, and the pooled ES was 2.0% (95% CI: 0.6%−3.8%). After excluding ten studies (23, 25–27, 31, 37, 39, 41, 43, 44), the *I*^2^ of new PPM implantation dropped to 26.1%, and the pooled ES was 10.2% (95% CI: 7.8%−12.8%). Overall, after excluding studies that exerted a significant impact on heterogeneity, the pooled ES for AKI exhibited a slight increase, whereas major vascular complications and major bleeding events showed a significant reduction, with minimal changes observed in all other indicators.

### Publication bias test

Tables 2, 3 present the results of the publication bias assessment for perioperative and 1-year post-operative outcomes. Because only two studies reported the 1-year incidence of AKI, the Egger test could not be performed for this outcome. For all other outcomes, the Egger test indicated no significant publication bias (*P* > 0.05).

## Discussion

TAVR was initially used primarily for the treatment of patients with pure severe aortic valve stenosis (11). In recent years, with device improvements and advances in clinical research, its use has expanded to patients with psNAVR who are at high surgical risk or have contraindications to surgery, providing a minimally invasive therapeutic alternative (45, 46). However, the success rate, mortality, and outcome-influencing factors of TAVR in psNAVR remain highly controversial. Therefore, this meta-analysis was conducted to evaluate the efficacy and safety of TAVR in patients with psNAVR using the most recent evidence. This analysis included 2,773 patients with psNAVR undergoing TAVR. We found that TAVR is a feasible treatment option for patients with psNAVR who are at high surgical risk. However, geographic location, surgical risk, valve type, and procedural approach influenced the incidence of adverse events after TAVR in psNAVR.

The main challenge in treating patients with psNAVR using TAVR is the absence of calcification in the device landing zone, which may lead to inadequate fixation of the prosthetic valve at the annulus during deployment and result in device displacement (13). In this study, the device success rate of TAVR for psNAVR was 87.5%, a satisfactory outcome consistent with the findings of Beerkens F J et al. (11), who reported a device success rate of 86.3% in patients with NAVR undergoing TAVR. It has also been reported that the operative mortality of patients with AR undergoing surgical aortic valve replacement is 3.4% (10). In comparison, although the perioperative all-cause mortality of psNAVR patients undergoing TAVR was only 3.1%, the 1-year all-cause mortality reached 9.3%. The higher mortality in psNAVR patients treated with TAVR is likely attributable to the fact that most patients included in this meta-analysis were at high surgical risk and often had depressed LVEF, concomitant severe mitral regurgitation, or an enlarged aortic diameter. Overall, TAVR appears to be a valuable therapeutic option for patients with psNAVR who are at high surgical risk.

The worsening of HF has been recognized as the most common cause of rehospitalization after TAVR (47). In this study, the incidences of perioperative adverse events such as stroke and myocardial infarction were relatively low; however, the 1-year rehospitalization rate due to HF reached 19%. This phenomenon may be attributable to multiple post-operative factors, including severe prosthesis–patient mismatch, significant paravalvular leaks, and conduction disturbances, all of which can increase the risk of HF. We also found that the 1-year incidences of AKI and new PPM implantation were close to 10%, which warrants clinical attention. AKI is a serious complication of TAVR and may result from several factors, including the transapical approach, sustained hypotension, and high contrast volume (48). In addition, several factors may contribute to adverse events after TAVR for psNAVR. For example, Witberg et al. (49) reported a significant association between center valve preference and late mortality. Similarly, Protasiewicz et al. (50) emphasized that when TAVR is performed with self-expanding valves at centers that predominantly favor balloon-expandable valves, 2-year mortality and periprocedural outcomes appear to be compromised. In this study, subgroup analyses showed that geographic location, surgical risk, valve type, and procedural approach were factors influencing the incidence of adverse events after TAVR for psNAVR. SEV combined with the TA are associated with the most favorable outcomes (highest device success, lowest overall adverse events); for patients at high risk of mortality, BEV may be preferred for their superior mortality reduction; SEV/BEV and multi-approach should be avoided due to significantly worse outcomes. In addition, geographic location is a surrogate for modifiable clinical and systemic factors (patient selection, procedural care, center expertise) rather than an independent risk factor. Its primary value is to highlight: (a) Targetable risks: western centers should prioritize bleeding/stroke prevention strategies proven effective in Chinese cohorts; (b) Contextualized risk stratification: adjust risk estimates based on regional practice patterns (e.g., higher bleeding risk in Western patients); (c) Quality improvement: standardize best practices (e.g., high-volume centers, personalized anticoagulation) across regions to reduce disparities. In clinical practice, geographic location should complement—not replace—objective risk scores and patient-specific factors (e.g., comorbidities, anatomy) to guide TAVR candidate selection and perioperative management. Also, subgroup analysis indicated that the generation of devices had significant impact on the major bleeding events and new PPM implantation. Therefore, when applying TAVR for psNAVR in clinical practice, these influencing factors should be carefully considered.

In this meta-analysis, the number of included studies was moderate, the cumulative sample size was large, and the pooled effect sizes were estimated with high precision. The methodological quality of the included studies was generally moderate, and the overall risk of bias was low. Importantly, no significant publication bias was detected, supporting the reliability of the results. However, several limitations should be considered. First, for outcomes other than all-cause mortality, relatively few studies reported 1-year post-operative data. Additional high-quality research is needed to confirm the stability and generalizability of these findings. Second, the methodological and clinical heterogeneity among the included studies was considerable, which contributed to substantial statistical heterogeneity in some outcomes. Although random-effects models were applied, interpretation of these results should remain cautious. Third, due to limited follow-up data, it was not possible to assess the medium- or long-term benefits of TAVR in psNAVR patients.

## Conclusion

In conclusion, this meta-analysis demonstrated that TAVR is a feasible treatment option for patients with psNAVR who are at high surgical risk. Geographic location, surgical risk, valve type, and procedural approach were identified as factors influencing the incidence of adverse events after TAVR in psNAVR. These findings provide an evidence-based reference to guide clinicians and healthcare teams in the management of patients with psNAVR.

## Data Availability

The original contributions presented in the study are included in the article/Supplementary material, further inquiries can be directed to the corresponding author.

## Supplementary material

Supplementary Figure S1 Forest plot of one-year all-cause mortality.

Supplementary Figure S2 Forest plot of one-year cardiovascular mortality.

Supplementary Figure S3 Forest plot of one-year stroke incidence.

Supplementary Figure S4 Forest plot of one-year acute kidney injury.

Supplementary Figure S5 Forest plot of one-year new-onset myocardial infarction.

Supplementary Figure S6 Forest plot of one-year major bleeding events.

Supplementary Figure S7 Forest plot of one-year readmission due to heart failure.

Supplementary Figure S8 Forest plot of one-year new permanent pacemaker implantation.

Supplementary Figure S9 Sensitivity analysis of device success of perioperative.

Supplementary Figure S10 Sensitivity analysis of all-cause mortality of perioperative.

Supplementary Figure S11 Sensitivity analysis of cardiovascular mortality of perioperative.

Supplementary Figure S12 Sensitivity analysis of stroke of perioperative.

Supplementary Figure S13 Sensitivity analysis of acute kidney injury of perioperative.

Supplementary Figure S14 Sensitivity analysis of major vascular complications of perioperative.

Supplementary Figure S15 Sensitivity analysis of major bleeding events of perioperative.

Supplementary Table S1 Search strategy and results for PubMed.

Supplementary Table S2 Search strategy and results for Embase.

Supplementary Table S3 Search strategy and results for the Cochrane Library.

Supplementary Table S4 Search strategy and results for Web of Science.

Supplementary Table S5 Methodological quality assessment of the included studies.

Supplementary Table S6 PRISMA_checklist.
