# Supplementary figures and images for: Efficacy and safety of transcatheter aortic valve replacement for the treatment of pure severe native aortic valve regurgitation: a single-arm meta-analysis
Source: Front Med (Lausanne). 2026 Mar 4;13:1735206. doi: 10.3389/fmed.2026.1735206 (PMC12996224; doi:10.3389/fmed.2026.1735206)

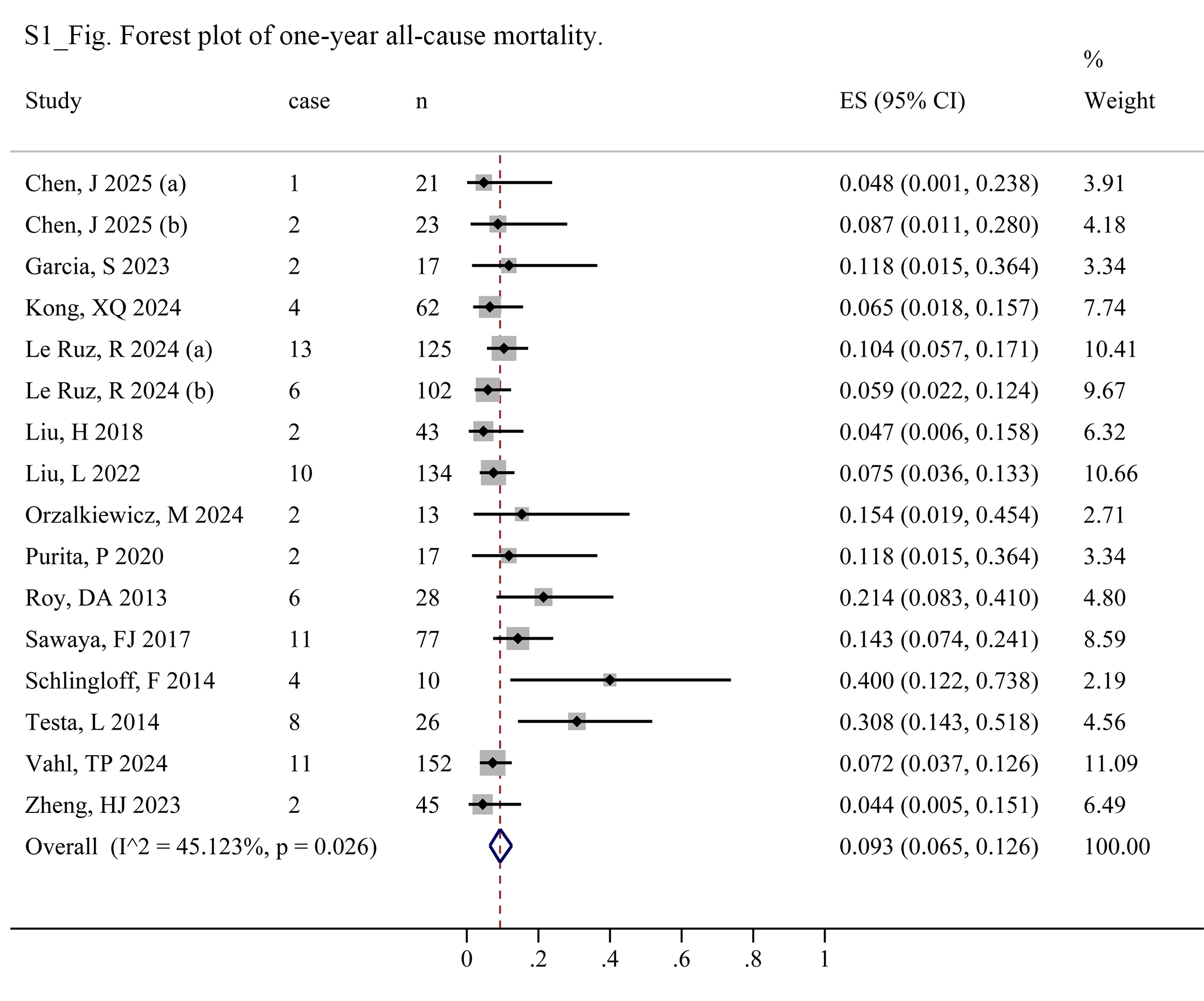

Supplement: Supplementary Figure S1 — Forest plot of one-year all-cause mortality. [file Image_1.tif]

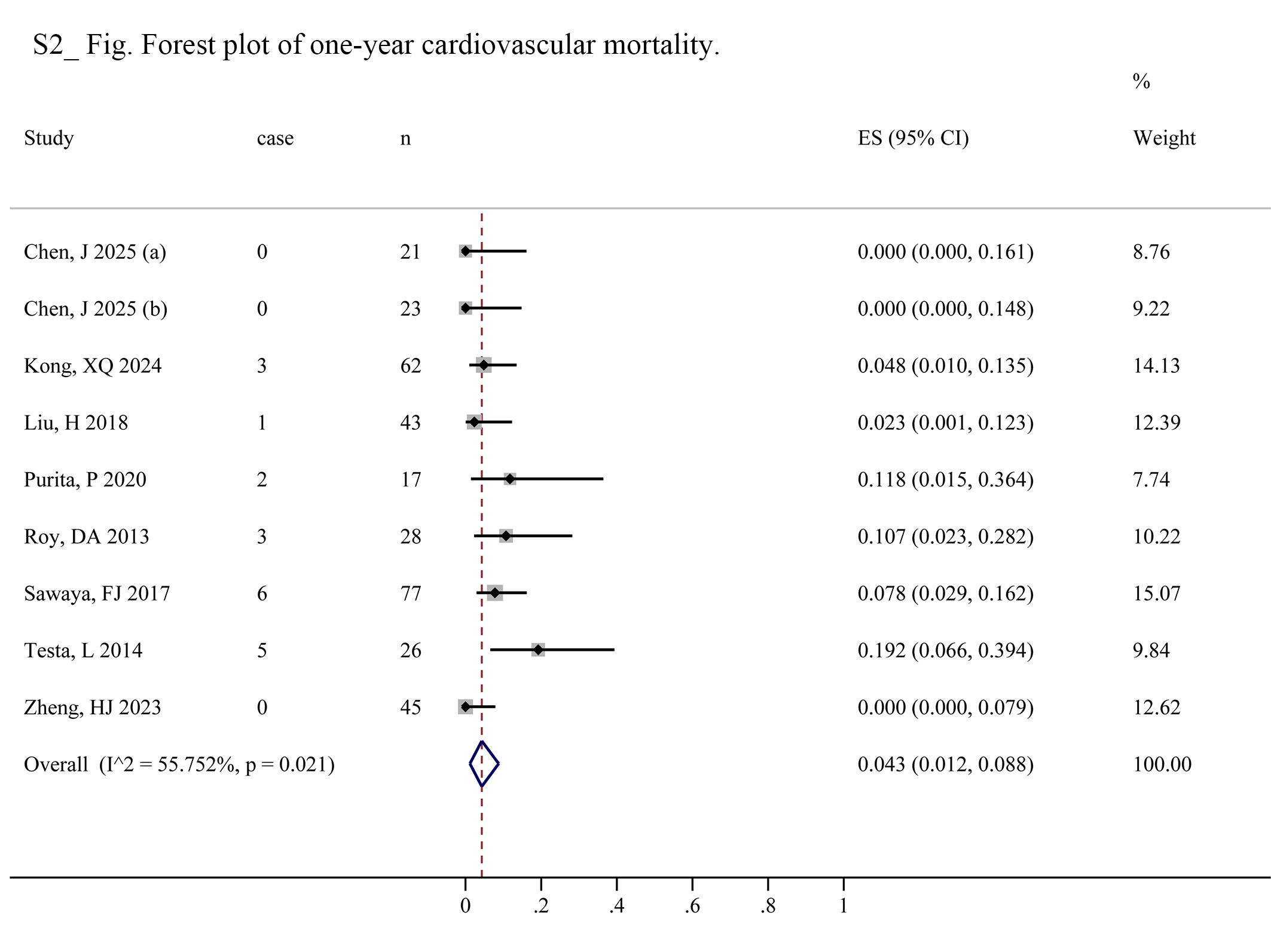

Supplement: Supplementary Figure S2 — Forest plot of one-year cardiovascular mortality. [file Image_2.tif]

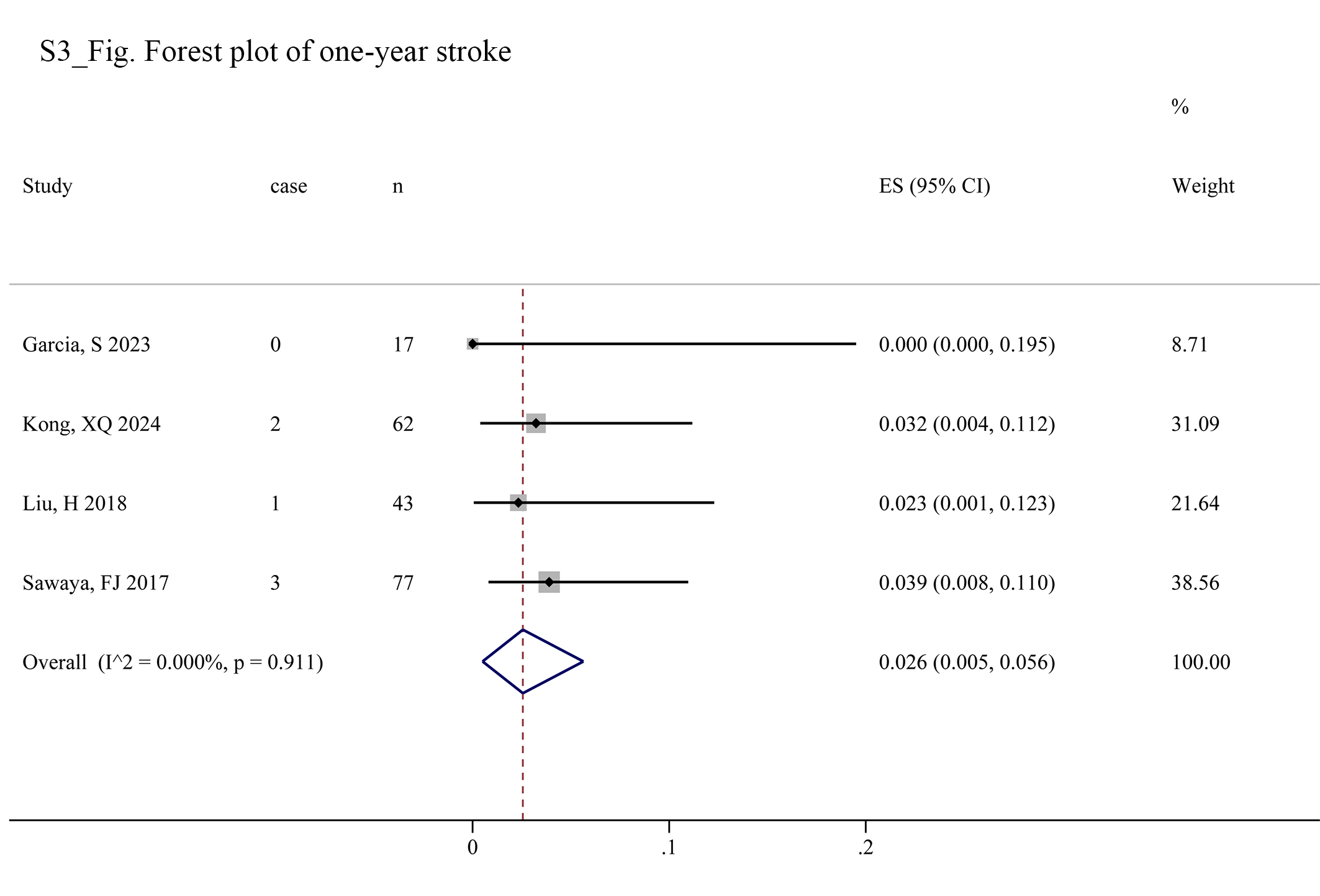

Supplement: Supplementary Figure S3 — Forest plot of one-year stroke incidence. [file Image_3.tif]

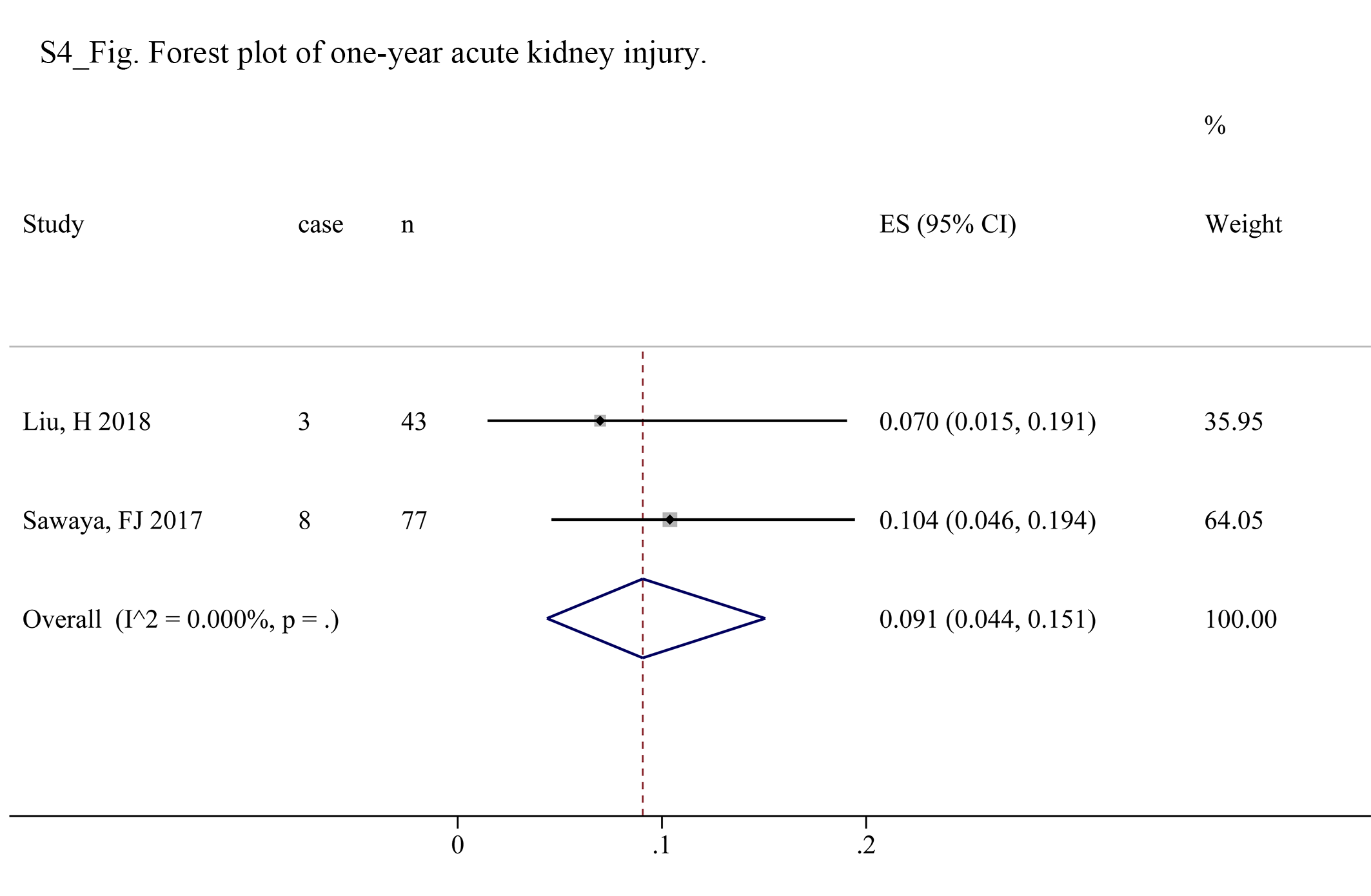

Supplement: Supplementary Figure S4 — Forest plot of one-year acute kidney injury. [file Image_4.tif]

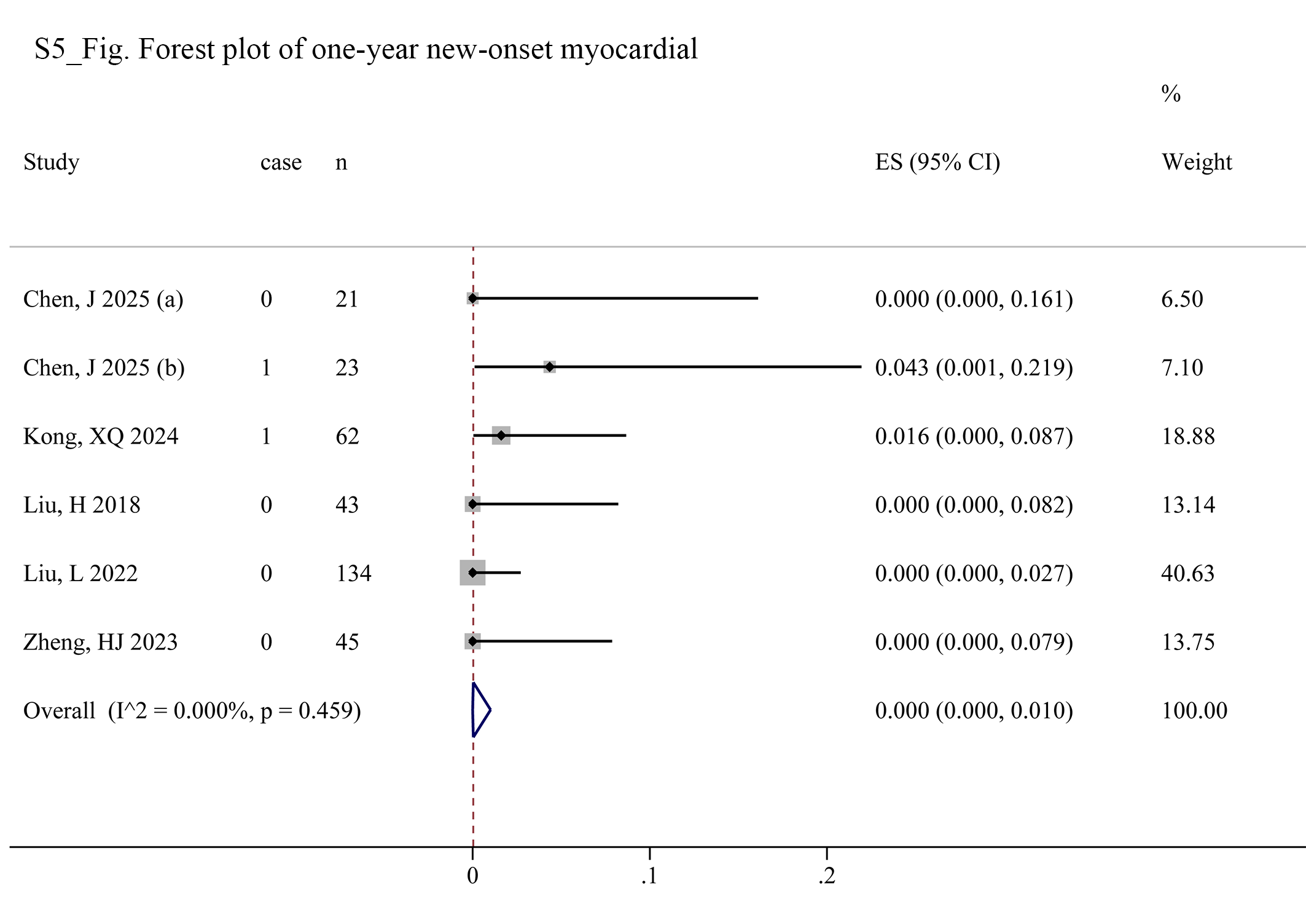

Supplement: Supplementary Figure S5 — Forest plot of one-year new-onset myocardial infarction. [file Image_5.tif]

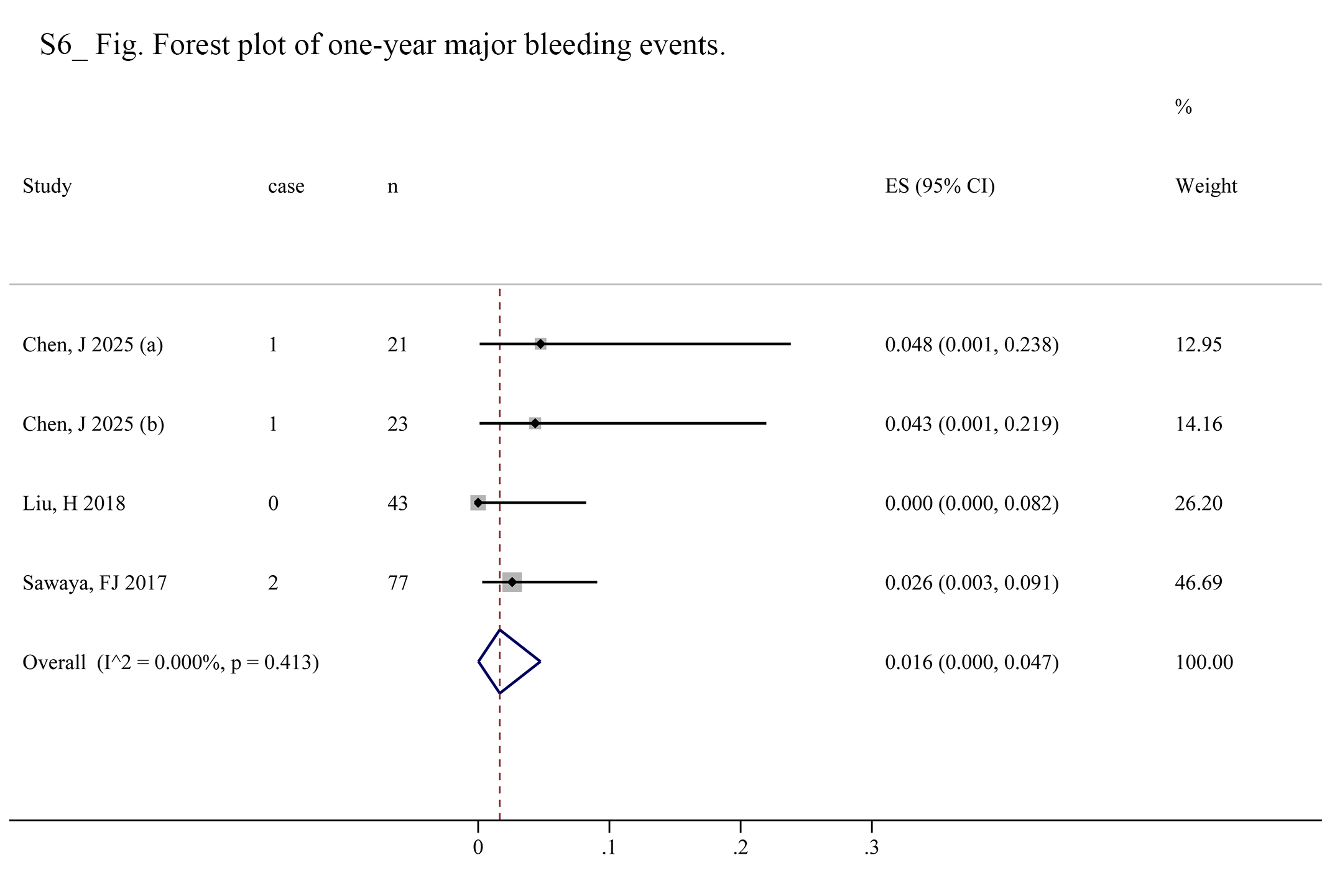

Supplement: Supplementary Figure S6 — Forest plot of one-year major bleeding events. [file Image_6.tif]

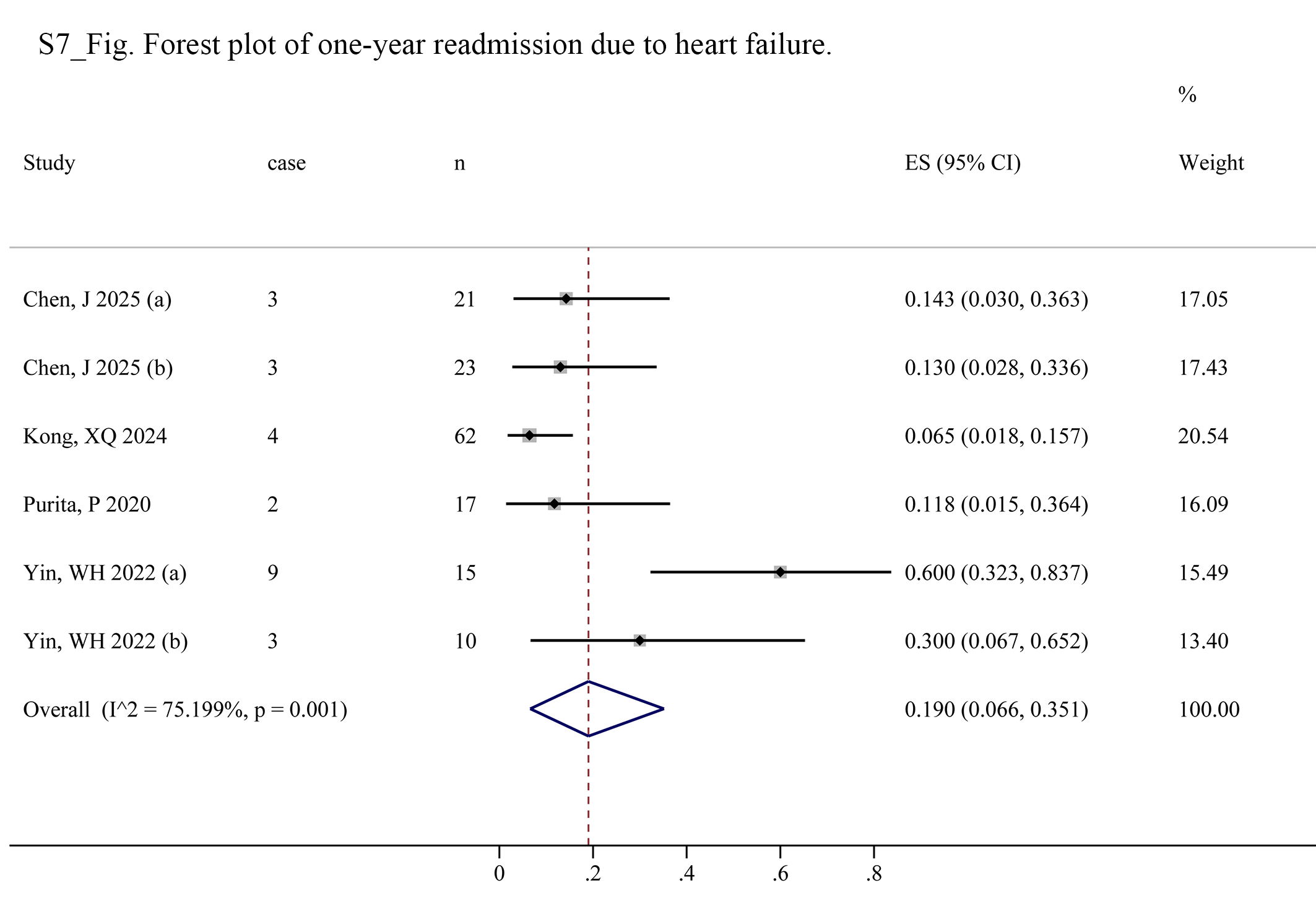

Supplement: Supplementary Figure S7 — Forest plot of one-year readmission due to heart failure. [file Image_7.tif]

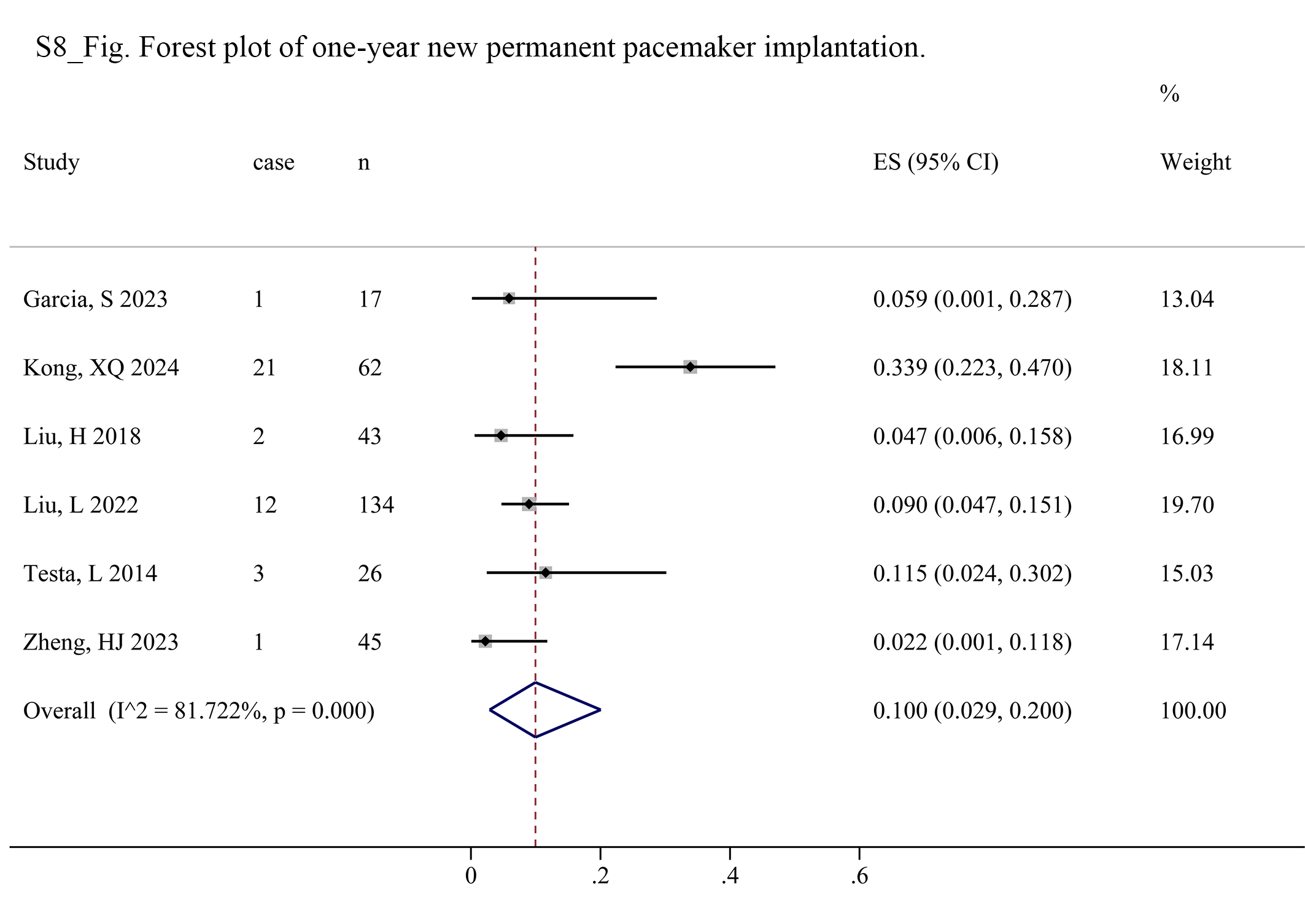

Supplement: Supplementary Figure S8 — Forest plot of one-year new permanent pacemaker implantation. [file Image_8.tif]

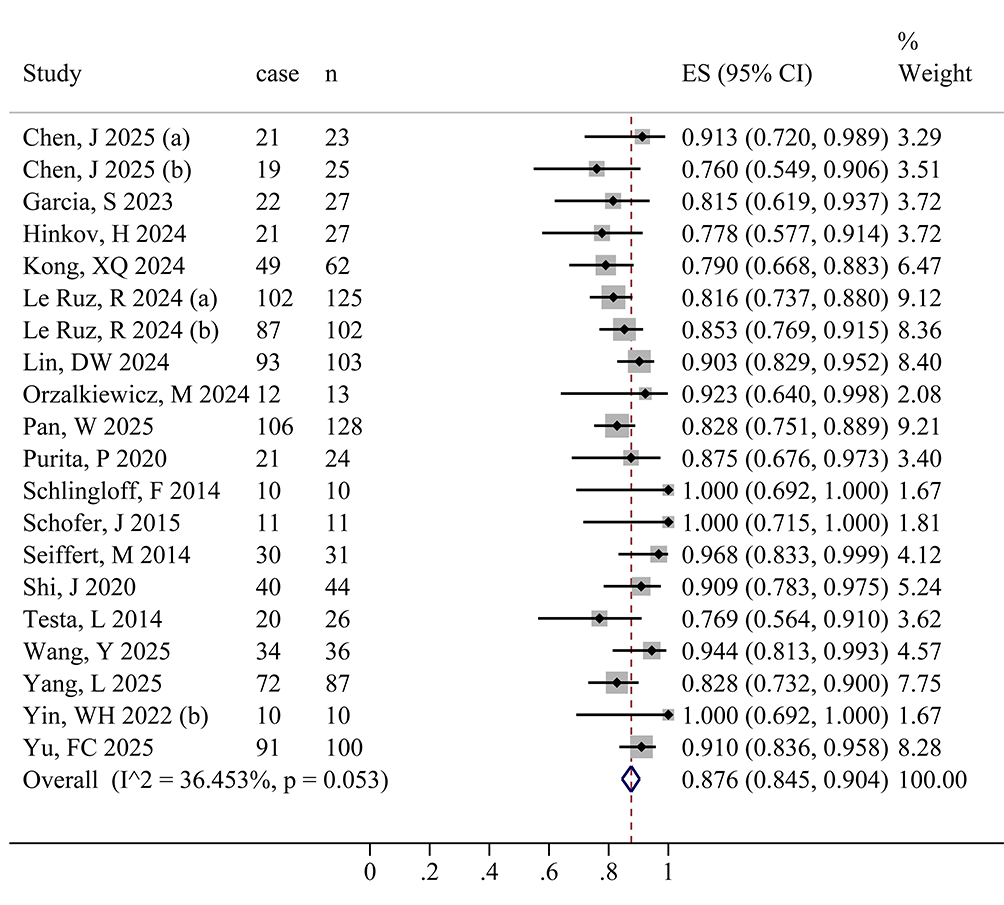

Supplement: Supplementary Figure S9 — Sensitivity analysis of device success of perioperative. [file Image_9.tif]

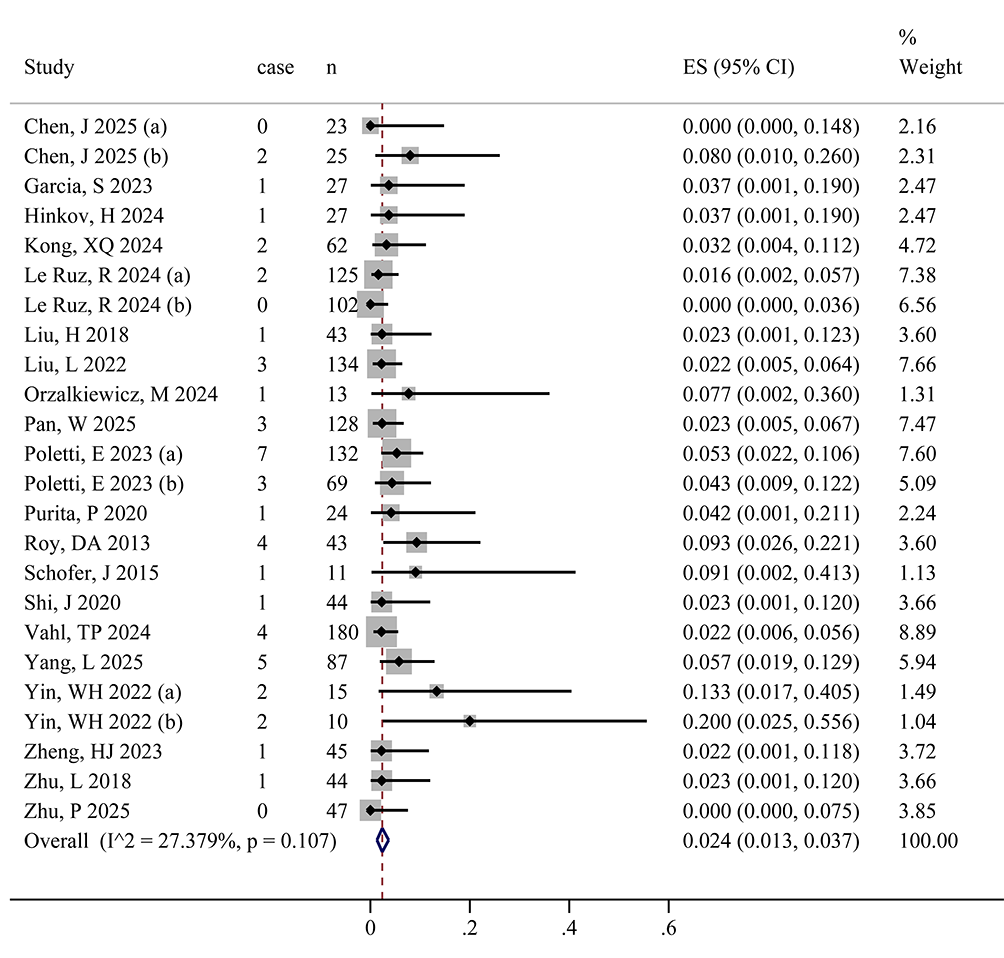

Supplement: Supplementary Figure S10 — Sensitivity analysis of all-cause mortality of perioperative. [file Image_10.tif]

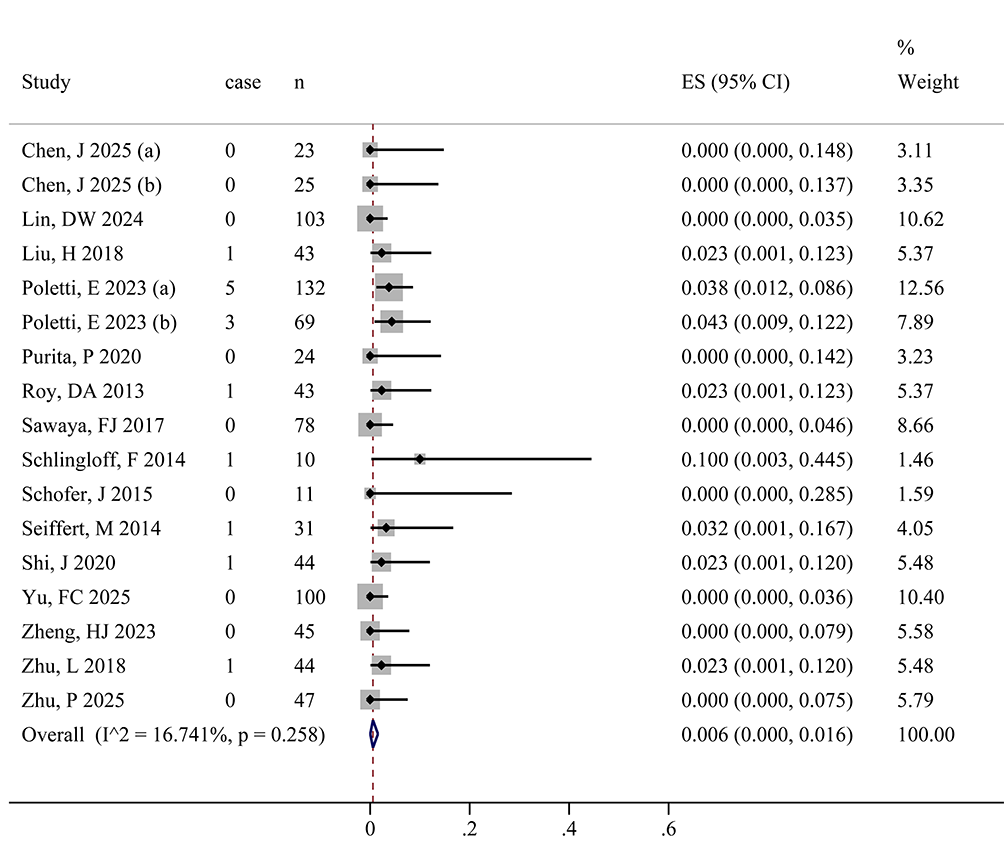

Supplement: Supplementary Figure S11 — Sensitivity analysis of cardiovascular mortality of perioperative. [file Image_11.tif]

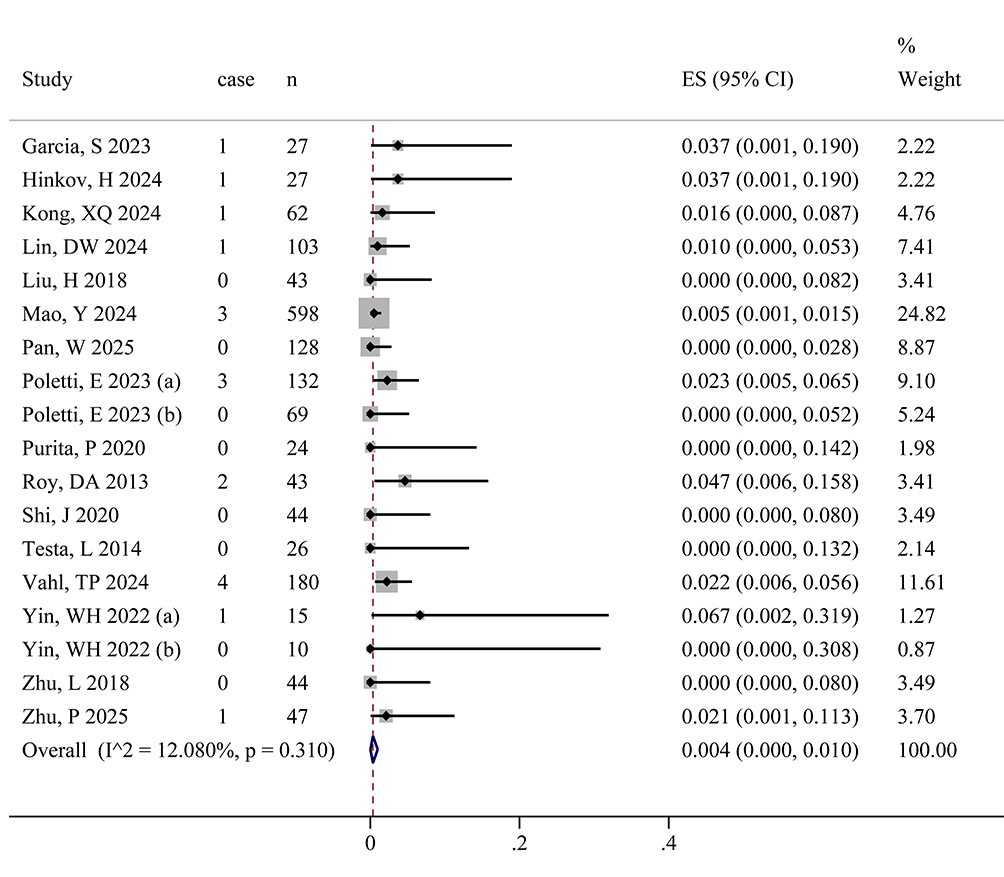

Supplement: Supplementary Figure S12 — Sensitivity analysis of stroke of perioperative. [file Image_12.tif]

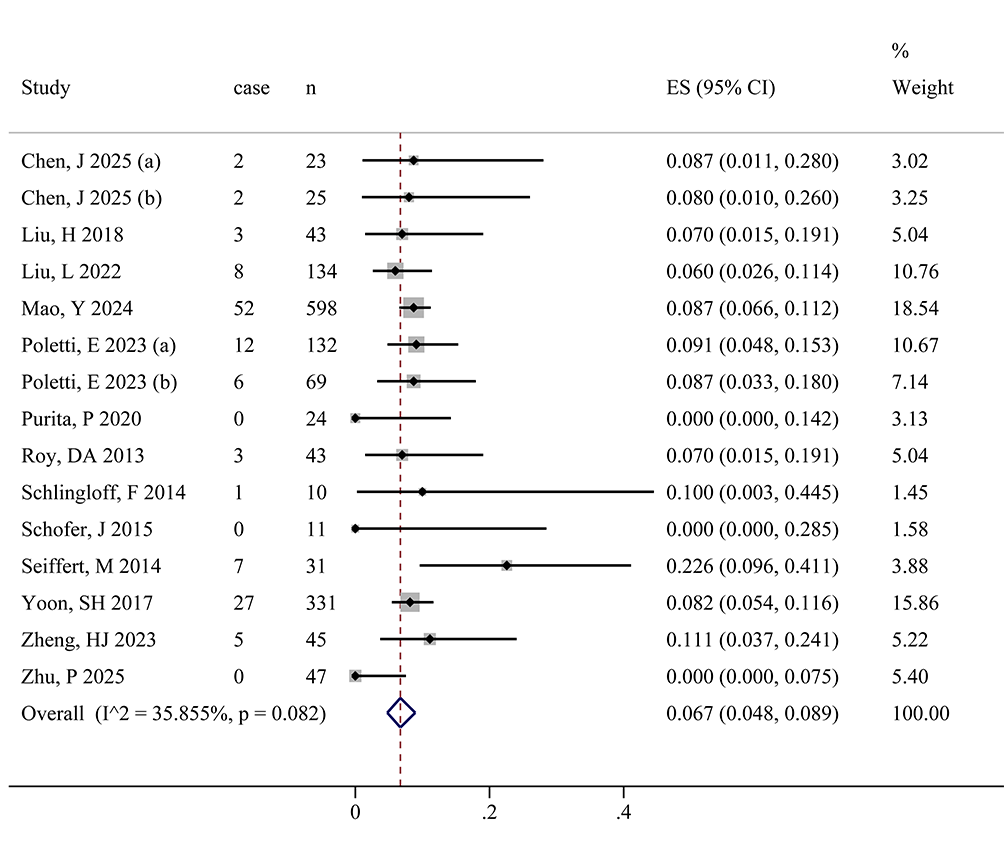

Supplement: Supplementary Figure S13 — Sensitivity analysis of acute kidney injury of perioperative. [file Image_13.tif]

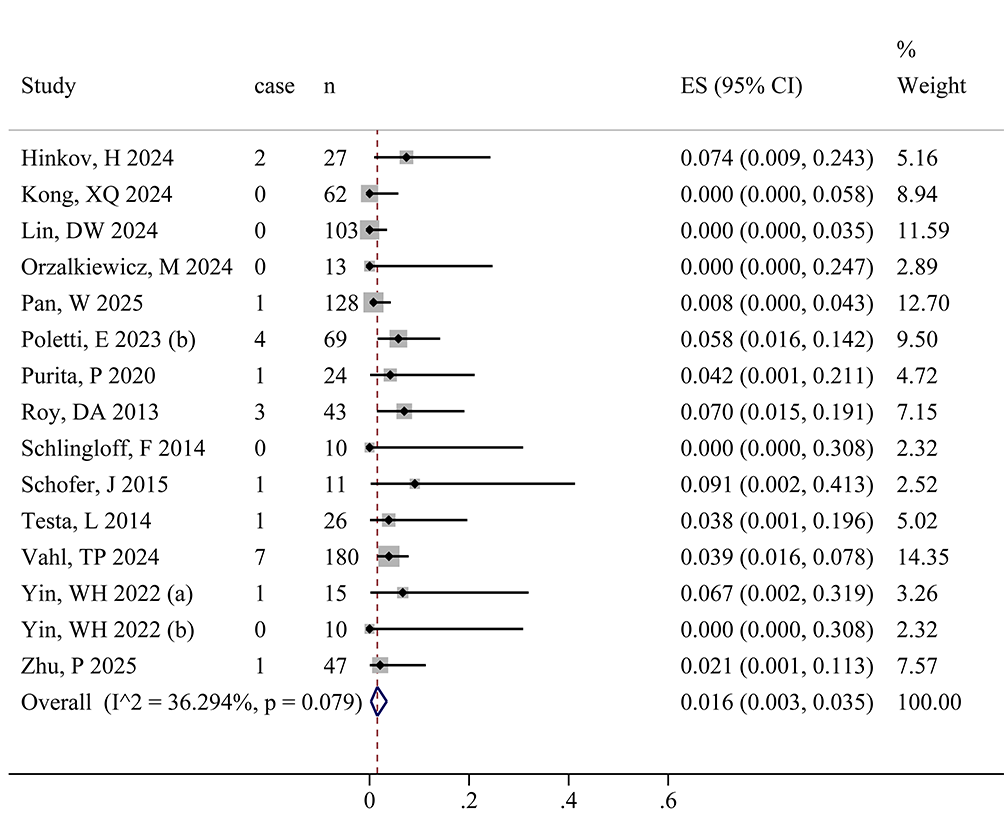

Supplement: Supplementary Figure S14 — Sensitivity analysis of major vascular complications of perioperative. [file Image_14.tif]

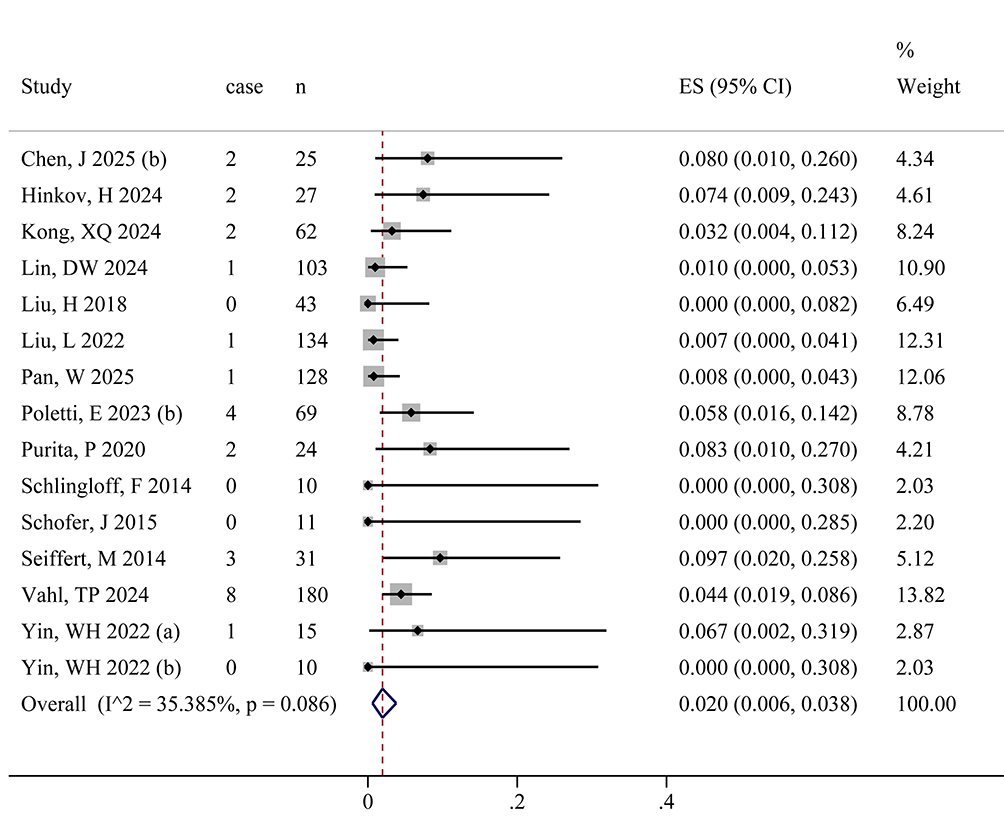

Supplement: Supplementary Figure S15 — Sensitivity analysis of major bleeding events of perioperative. [file Image_15.tif]
