# Supplementary material for: Efficacy and safety of transcatheter aortic valve replacement for the treatment of pure severe native aortic valve regurgitation: a single-arm meta-analysis
Source: Front Med (Lausanne). 2026 Mar 4;13:1735206. doi: 10.3389/fmed.2026.1735206 (PMC12996224; doi:10.3389/fmed.2026.1735206)
Supplement: Supplementary Table S1 — Search strategy and results for PubMed. [file Table_1.docx]

**Supplementary Table 1.** **Search Process and Results of PubMed.**

| Search | Query | Items found |
| --- | --- | --- |
| #1 | "aortic valve insufficiency"[MeSH Terms] OR "aortic valve insufficiency"[All Fields] | 16925 |
| #2 | "aortic valve regurgitation"[All Fields] | 1242 |
| #3 | "aortic regurgitation"[All Fields] | 10302 |
| #4 | "pure"[All Fields] | 221565 |
| #5 | (#1 OR #2 OR #3) AND #4 | 539 |
| #6 | "transcatheter aortic valve replacement"[MeSH Terms] OR "transcatheter aortic valve replacement"[All Fields] | 17025 |
| #7 | "transcatheter aortic valve implantation"[All Fields] | 9170 |
| #8 | "TAVR"[All Fields] OR "TAVI"[All Fields] | 15461 |
| #9 | #6 OR #7 OR #8 | 22274 |
| #10 | #5 AND #9 | 212 |
