# Supplementary material for: Efficacy and safety of transcatheter aortic valve replacement for the treatment of pure severe native aortic valve regurgitation: a single-arm meta-analysis
Source: Front Med (Lausanne). 2026 Mar 4;13:1735206. doi: 10.3389/fmed.2026.1735206 (PMC12996224; doi:10.3389/fmed.2026.1735206)
Supplement: Supplementary Table S2 — Search strategy and results for Embase. [file Table_2.docx]

**Supplementary Table 2. Search Process and Results of Embase.**

| Search | Query | Items found |
| --- | --- | --- |
| #1 | ('aortic valve insufficiency'/exp OR 'aortic valve insufficiency' OR 'aortic valve regurgitation'/exp OR 'aortic valve regurgitation' OR 'aortic regurgitation'/exp OR 'aortic regurgitation') | 35893 |
| #2 | ('pure'/exp OR pure) | 210315 |
| #3 | #1 AND #2 | 712 |
| #4 | (tavr OR 'transcatheter aortic valve replacement'/exp OR 'transcatheter aortic valve replacement' OR 'transcatheter aortic valve implantation'/exp OR 'transcatheter aortic valve implantation' OR 'tavi'/exp OR tavi) | 43356 |
| #5 | #3 AND #4 | 345 |
