# Supplementary material for: Efficacy and safety of transcatheter aortic valve replacement for the treatment of pure severe native aortic valve regurgitation: a single-arm meta-analysis
Source: Front Med (Lausanne). 2026 Mar 4;13:1735206. doi: 10.3389/fmed.2026.1735206 (PMC12996224; doi:10.3389/fmed.2026.1735206)
Supplement: Supplementary Table S3 — Search strategy and results for the Cochrane Library. [file Table_3.docx]

**Supplementary Table 3. Search Process and Results of The Cochrane library.**

| Search | Query | Items found |
| --- | --- | --- |
| #1 | MeSH descriptor: [Aortic Valve Insufficiency] explode all trees | 200 |
| #2 | ((aortic valve insufficiency) OR (aortic valve regurgitation) OR (aortic regurgitation)):ti,ab,kw (Word variations have been searched) | 894 |
| #3 | #1 OR #2 | 894 |
| #4 | (pure):ti,ab,kw (Word variations have been searched) | 7630 |
| #5 | #3 AND #4 | 13 |
| #6 | MeSH descriptor: [Transcatheter Aortic Valve Replacement] explode all trees | 550 |
| #7 | (TAVR OR (transcatheter aortic valve replacement) OR (transcatheter aortic valve implantation) OR TAVI):ti,ab,kw (Word variations have been searched) | 1649 |
| #8 | #6 OR #7 | 1649 |
| #9 | #5 AND #8 | 5 |
| #10 | #9 in Trials | 5 |
