# Supplementary material for: Efficacy and safety of transcatheter aortic valve replacement for the treatment of pure severe native aortic valve regurgitation: a single-arm meta-analysis
Source: Front Med (Lausanne). 2026 Mar 4;13:1735206. doi: 10.3389/fmed.2026.1735206 (PMC12996224; doi:10.3389/fmed.2026.1735206)
Supplement: Supplementary Table S4 — Search strategy and results for Web of Science. [file Table_4.docx]

**Supplementary Table 4. Search Process and Results of Web of Science.**

| Search | Query | Items found |
| --- | --- | --- |
| #1 | pure (All Fields) | 833548 |
| #2 | (aortic valve insufficiency) OR (aortic valve regurgitation) OR (aortic regurgitation) (All Fields) | 20660 |
| #3 | TAVR OR (transcatheter aortic valve replacement) OR (transcatheter aortic valve implantation) OR TAVI (All Fields) | 30304 |
| #4 | #1 AND #2 AND #3 | 251 |
