# Supplementary material for: Efficacy and safety of transcatheter aortic valve replacement for the treatment of pure severe native aortic valve regurgitation: a single-arm meta-analysis
Source: Front Med (Lausanne). 2026 Mar 4;13:1735206. doi: 10.3389/fmed.2026.1735206 (PMC12996224; doi:10.3389/fmed.2026.1735206)
Supplement: Supplementary Table S5 — Methodological quality assessment of the included studies. [file Table_5.docx]

**Supplementary Table 5. Quality assessment of the included studies.**

| Study | A | B | C | D | E | F | G | H | Total Scores |
| --- | --- | --- | --- | --- | --- | --- | --- | --- | --- |
| Chen, J 2025 | 2 | 1 | 0 | 2 | 2 | 2 | 2 | 0 | 11 |
| Garcia, S 2023 | 2 | 1 | 0 | 2 | 2 | 2 | 1 | 0 | 10 |
| Hinkov, H 2024 | 2 | 1 | 0 | 2 | 2 | 2 | 2 | 0 | 11 |
| Kong, XQ 2024 | 2 | 2 | 0 | 2 | 2 | 2 | 2 | 0 | 12 |
| Le Ruz, R 2024 | 2 | 2 | 2 | 2 | 2 | 0 | 2 | 0 | 12 |
| Lin, DW 2024 | 2 | 2 | 0 | 1 | 2 | 0 | 2 | 0 | 9 |
| Liu, H 2018 | 2 | 1 | 0 | 2 | 2 | 2 | 2 | 0 | 11 |
| Liu, L 2022 | 2 | 1 | 0 | 2 | 2 | 2 | 2 | 0 | 11 |
| Mao, Y 2024 | 2 | 2 | 0 | 2 | 2 | 2 | 2 | 0 | 12 |
| Orzalkiewicz, M 2024 | 2 | 2 | 0 | 2 | 2 | 2 | 2 | 0 | 12 |
| Pan, W 2025 | 2 | 1 | 2 | 2 | 2 | 0 | 2 | 0 | 11 |
| Poletti, E 2023 | 2 | 1 | 0 | 2 | 2 | 2 | 2 | 0 | 11 |
| Purita, P 2020 | 2 | 2 | 0 | 2 | 2 | 2 | 1 | 0 | 11 |
| Roy, DA 2013 | 2 | 1 | 0 | 2 | 2 | 2 | 1 | 0 | 10 |
| Sawaya, FJ 2017 | 2 | 1 | 0 | 2 | 2 | 2 | 2 | 0 | 11 |
| Schlingloff, F 2014 | 2 | 1 | 0 | 2 | 2 | 2 | 2 | 0 | 11 |
| Schofer, J 2015 | 2 | 1 | 0 | 2 | 2 | 0 | 2 | 0 | 9 |
| Seiffert, M 2014 | 2 | 1 | 0 | 2 | 2 | 0 | 2 | 0 | 9 |
| Shi, J 2020 | 2 | 1 | 2 | 2 | 2 | 2 | 2 | 0 | 13 |
| Testa, L 2014 | 2 | 2 | 0 | 2 | 2 | 2 | 2 | 0 | 12 |
| Vahl, TP 2024 | 2 | 2 | 2 | 2 | 2 | 2 | 1 | 2 | 15 |
| Wang, Y 2025 | 2 | 2 | 2 | 2 | 2 | 2 | 2 | 0 | 14 |
| Yang, L 2025 | 2 | 1 | 0 | 2 | 2 | 0 | 2 | 0 | 9 |
| Yin, WH 2022 | 2 | 2 | 0 | 2 | 2 | 2 | 2 | 0 | 12 |
| Yoon, SH 2017 | 2 | 2 | 0 | 2 | 2 | 2 | 2 | 0 | 12 |
| Yu, FC 2025 | 2 | 1 | 2 | 2 | 2 | 2 | 2 | 0 | 13 |
| Zheng, HJ 2023 | 2 | 2 | 0 | 2 | 2 | 2 | 2 | 0 | 12 |
| Zhu, L 2018 | 2 | 1 | 0 | 2 | 2 | 1 | 2 | 0 | 10 |
| Zhu, P 2025 | 2 | 2 | 0 | 2 | 2 | 1 | 2 | 0 | 11 |

A: A clearly stated aim;

B: Inclusion of consecutive patients;

C: Prospective collection of data;

D: Endpoints appropriate to the aim of the study;

E: Unbiased assessment of the study endpoint;

F: Follow-up period appropriate to the aim of the study;

G: Loss to follow up less than 5%;

H: Prospective calculation of the study size.
